# High-throughput data and modeling reveal insights into the mechanisms of cooperative DNA-binding by transcription factor proteins

**DOI:** 10.1093/nar/gkad872

**Published:** 2023-10-27

**Authors:** Vincentius Martin, Farica Zhuang, Yuning Zhang, Kyle Pinheiro, Raluca Gordân

**Affiliations:** Department of Computer Science, Durham, NC 27708, USA; Center for Genomic & Computational Biology, Durham, NC 27708, USA; Department of Computer Science, Durham, NC 27708, USA; Center for Genomic & Computational Biology, Durham, NC 27708, USA; Center for Genomic & Computational Biology, Durham, NC 27708, USA; Program in Computational Biology & Bioinformatics, Durham, NC 27708, USA; Department of Computer Science, Durham, NC 27708, USA; Center for Genomic & Computational Biology, Durham, NC 27708, USA; Department of Computer Science, Durham, NC 27708, USA; Center for Genomic & Computational Biology, Durham, NC 27708, USA; Department of Biostatistics & Bioinformatics, Department of Molecular Genetics and Microbiology, Department of Cell Biology, Duke University, Durham, NC 27708, USA

## Abstract

Cooperative DNA-binding by transcription factor (TF) proteins is critical for eukaryotic gene regulation. In the human genome, many regulatory regions contain TF-binding sites in close proximity to each other, which can facilitate cooperative interactions. However, binding site proximity does not necessarily imply cooperative binding, as TFs can also bind independently to each of their neighboring target sites. Currently, the rules that drive cooperative TF binding are not well understood. In addition, it is oftentimes difficult to infer direct TF–TF cooperativity from existing DNA-binding data. Here, we show that *in vitro* binding assays using DNA libraries of a few thousand genomic sequences with putative cooperative TF-binding events can be used to develop accurate models of cooperativity and to gain insights into cooperative binding mechanisms. Using factors ETS1 and RUNX1 as our case study, we show that the distance and orientation between ETS1 sites are critical determinants of cooperative ETS1–ETS1 binding, while cooperative ETS1–RUNX1 interactions show more flexibility in distance and orientation and can be accurately predicted based on the affinity and sequence/shape features of the binding sites. The approach described here, combining custom experimental design with machine-learning modeling, can be easily applied to study the cooperative DNA-binding patterns of any TFs.

## Graphical Abstract

**Graphical Abstract:**
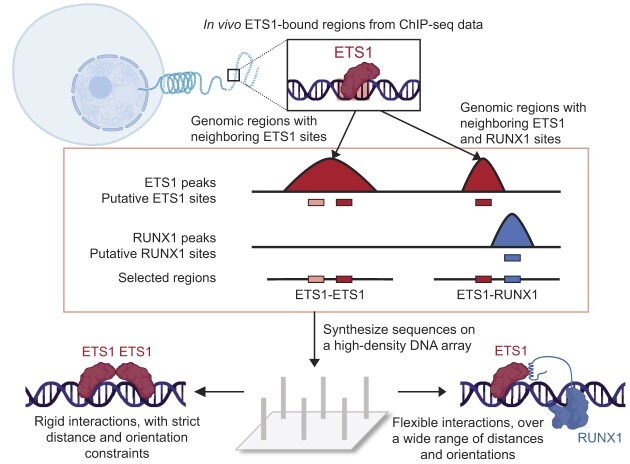

## Introduction

Transcription factors bind to DNA in a sequence-specific manner, a key process in the regulation of gene expression. In human cells, TF binding sites are oftentimes present in clusters, i.e. multiple sites located in close proximity to each other (1,2). Clusters of binding sites recognized by the same TF (i.e. homotypic clusters) occur in >50% of human promoters and are especially abundant in developmental enhancers (1–3). During evolution, clustering of TF binding sites provides a mechanism for achieving high-affinity binding regions while buffering against single detrimental mutations that would completely abolish binding (4). Clusters of sites bound by different TFs (i.e. heterotypic clusters) are critical for combinatorial regulation, which is especially important in complex multicellular organisms (5). In addition, given that most eukaryotic TFs have short motifs that are insufficient to specify unique target sites across the genome, heterotypic clustering of binding sites helps increase target specificity by using combinations of short motifs (5).

Mechanistically, binding sites located in close proximity to each other allow TFs to bind cooperatively, thus stabilizing the binding and enhancing each TF’s contribution to gene regulation (6). Sites that are likely bound cooperatively *in vivo* are oftentimes bound weakly when only individual TFs are present in the cell (7). Thus, cooperative interactions serve to enhance the overall DNA-binding affinity of the TF–TF complex and enable the utilization of low-affinity binding sites in gene regulation. Cooperativity is also used to achieve specificity within TF families, where paralogs with similar DNA binding preferences may interact with different cofactors to regulate different sets of genes (8,9). Moreover, cooperativity has been used to explain the rapid rate of evolution of TF binding sites in multicellular organisms (10), by facilitating the emergence of shorter sites with low to medium affinity. This shows that cooperativity is an indispensable aspect in the regulation of gene expression in higher organisms.

Characterizing the cooperative DNA-binding patterns of TFs is not trivial. Even when two binding sites are located in close proximity in the genome, cooperative binding is not necessarily implied, as TFs can also bind independently to each of their target sites. High-throughput assays based on Systematic Evolution of Ligands by Exponential Enrichment (SELEX) have been used, on a large scale, to identify composite motifs that reflect orientation and spacing preferences for cooperative TF binding at neighboring sites (11,12). In these studies, at most three composite motifs were identified per TF (11) or pair of TFs (12), leaving open the question: at spacings and orientations for which composite motifs were not identified by SELEX-based methods, can cooperative binding events still occur? In addition, even at the preferred spacing/orientation between neighboring sites, are all binding events cooperative, or can TFs also bind independently? Finally, what features other than spacing and orientation are important for cooperative TF binding?

To address these questions, we developed an experimental approach to distinguish between cooperative and independent binding of TFs to neighboring DNA sites in high throughput. We used on-chip assays based on the genomic-context protein-binding microarray (gcPBM) technology (13,14), but with DNA libraries designed specifically to assess cooperative TF binding at sites located in close proximity in the genome. To develop and evaluate our approach, we used as a case study human TF ETS1, which is known to bind cooperatively with itself as well as with other TFs (15–25), including RUNX1 (also known as CBFα2, AML1 or PEBP2) (26–31). Studying both ETS1–ETS1 and ETS1–RUNX1 binding to DNA enabled us to compare mechanisms of ETS1 cooperative binding with different partners. In addition, existing in-depth knowledge on ETS1 cooperativity, from low-throughput structural, biochemical and molecular biology studies allowed us to validate the findings from our high-throughput approach.

## Materials and methods

### Identification of neighboring binding sites in the genome

**Figure 1.**
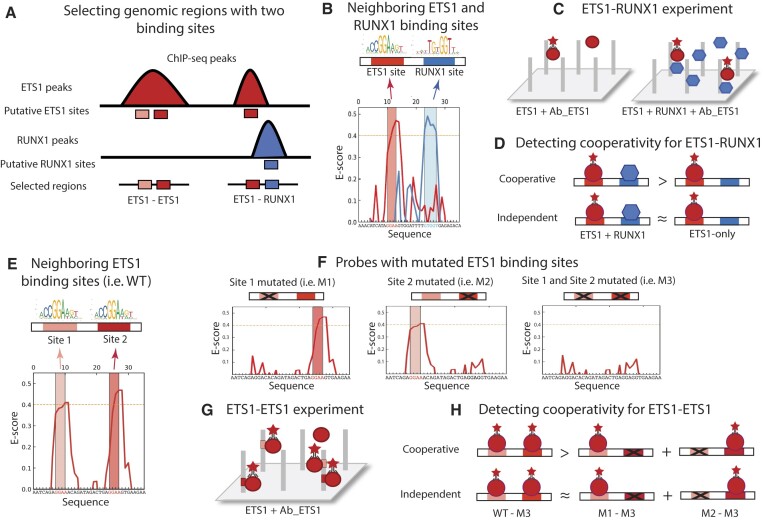
Experimental approach to detect cooperative TF binding. (**A**) Neighboring ETS1–ETS1 and ETS1–RUNX1 sites were identified within ChIP-seq peaks. (**B**) Example of a 36-bp genomic sequence with neighboring ETS1 (red) and RUNX1 (blue) sites. The sites were identified using universal PBM enrichment scores (E-scores) (34) and PWM models. PWM logos for ETS1 and RUNX1 are shown. The core base pairs in each binding site are highlighted in color. The dotted orange line denotes the *E*-score cutoff, 0.4, for calling specific TF binding sites (13,14). (**C**) Cooperative gcPBM experiment designed to detect cooperative binding events by comparing binding of ETS1 alone vs. in the presence of a high concentration of cooperator TF, RUNX1. Ab_ETS1: anti-ETS1 antibody. (**D**) Binding of ETS1–RUNX1 is considered cooperative when the ETS1 binding signal is significantly higher in the presence vs. the absence of RUNX1. (**E**) Example of a 36-bp genomic sequence with neighboring ETS1 sites. (**F**) DNA probes that represent mutants of the wild-type (WT) sequence with neighboring ETS1 sites from panel E. (**G**) Cooperative gcPBM experiment designed to detect cooperative binding events for the ETS1–ETS1 system, based on ETS1 binding measurements for the WT, M1, M2 and M3 probes. (**H**) Binding of ETS1 is considered cooperative when the signal intensity of the WT sequences is higher than the sum of the intensities of the individual sites, adjusted for non-specific binding (M3).

We used chromatin immunoprecipitation coupled with high-throughput sequencing (ChIP-seq) data from ENCODE (32) and Cistrome (33) (Supplementary Table S1) to identify genomic regions containing putative ETS1 binding sites in close proximity (4–24 bp) to other ETS1 sites or to RUNX1 binding sites, where the TFs might bind cooperatively (Figure 1A). For each available ETS1 ChIP-seq data set, we scanned the peaks using position weight matrix (PWM) models and *in vitro* binding data, as detailed below, to identify potential binding sites. As expected, we found that peaks containing multiple binding sites typically have higher ChIP-seq signal than peaks with single binding sites (Supplementary Figure S1). When comparing the ChIP-seq peaks of ETS1 and RUNX1, we found that RUNX1 peaks were oftentimes in close proximity to ETS1 ChIP-seq peaks (Supplementary Figure S2), consistent with cooperative binding between the two TFs (29,30). We used the ETS1 peaks that overlap RUNX1 peaks to identify potential sites of ETS1–RUNX1 cooperativity (Figure 1A). Importantly, in this study we focused on genomic rather than artificial DNA sequences because genomic regions represent sequences that the TFs encounter in the cell. Thus, we expect that the relevant determinants for cooperative vs. independent TF binding will be present in these genomic regions, and thus in our dataset.

### Design of DNA library for cooperative binding assay

We adapted the chip-based gcPBM assay (13) to measure cooperative and independent ETS1–ETS1 and ETS1–RUNX1 binding to thousands of genomic regions. In our original gcPBM assay, only individual TF binding sites were tested, using DNA libraries containing 36-bp genomic sequences centered on putative binding sites for the TF of interest (13,14). Here, the 36-bp probes in our DNA library are designed to contain genomic sequences with two binding sites, selected from ChIP-seq peaks (Figure 1A). We focused on sites located at a distance of 4–24 bp, calculated between the centers of the binding sites. Smaller distances were not considered, as the cores of the binding sites (i.e. the regions where DNA bases make direct contacts with the proteins) would be overlapping. Distances larger than 24 bp could not be tested due to the length limitation for the probes in our DNA library.

Putative TF binding sites were identified using PWM models and enrichment scores (E-scores) from universal PBM experiments (34). As in previous work, we searched for two consecutive 8-mers with *E*-score >0.4 (which generally indicates specific TF binding) (13,14), and we aligned the putative binding sites using PWMs (Figure 1B, E). Using this strategy, we identified 5093 neighboring ETS1–ETS1 sites and 3197 ETS1–RUNX1 sites. For each region, we designed a 36-bp DNA probe centered on the middle position(s) between the two sites. As in previous work (13,14,35), our DNA library also included negative control probes that lacked TF binding sites (400 for ETS1–RUNX1, 257 for ETS1–ETS1).

To allow the identification of ETS1–ETS1 cooperative binding events, our DNA library also contained, for each genomic region with two neighboring ETS1 sites, probes where one or both of the sites were mutated (Figure 1F, Supplementary Figure S3). Similar probes were added to the ETS1–RUNX1 library, with either the ETS1 or the RUNX1 site mutated; in this case, the mutated probes were used to normalize the binding data between different experiments, as described below.

### Cooperative gcPBM assay

The designed DNA sequences were synthesized as single-stranded molecules on high-density DNA microarrays (Agilent) in 4 × 180k format, with three or four replicate spots per sequence for the ETS1–ETS1 and ETS1–RUNX1 neighboring sites, respectively. The arrays were double-stranded and the protein-binding steps were performed following standard PBM protocols (34). Briefly, the double-stranded DNA libraries were incubated with the proteins of interest (ETS1, RUNX1 or ETS1 + RUNX1), and the level of bound protein at each spot was measured using primary antibodies specific for either ETS1 (Cell Signaling Technology 14069) or RUNX1 (Abcam ab23980) followed by Alexa488-conjugated secondary antibody (Invitrogen A-21206). Full-length, recombinant human ETS1 and RUNX1 proteins were expressed and purified as previously described (14). The arrays were scanned using a GenePix 4400A microarray scanner (Molecular Devices), and the fluorescence intensity at each DNA spot was used to represent the TF binding level for the DNA sequence at that spot. As shown in previous work (14,36,37), the PBM fluorescence intensities correlate remarkably well with independently measured equilibrium dissociation constants, and can be interpreted as the levels of bound TF at each of the sequences in our DNA library.

### Identification of ETS1–RUNX1 cooperative versus independent binding events

To identify cooperative binding events at the genomic regions containing neighboring ETS1–RUNX1 sites, we first measured ETS1 binding in the presence vs. absence of saturating levels of RUNX1 (Figure 1C), similarly to previous work (28). For ETS1 we used a concentration of 1 nM, selected because it resulted in a broad range of binding signals on our DNA arrays (Supplementary Table S2A; Supplementary Figure S4). For RUNX1 we chose a high concentration (25 nM) in order to ensure that, even at spots with low-affinity RUNX1 sites, a large fraction of the DNA molecules in the spots were bound.

Next, we compared the ETS1 binding signals in the ETS1-only versus the ETS1+RUNX1 experiments (Figure 1D). If the latter signal was significantly higher (p-value <0.02, one-sided Mann–Whitney *U* test applied to the four replicates spots), then we labelled the binding as ‘cooperative’. If the signals from the two experiments were similar (*P*-value ≥ 0.1), then we labelled the binding as ‘independent’. Sequences with intermediate *P*-values were considered ambiguous and were not included in classification analyses. The *P*-value cutoffs were determined based on the possible values for the Mann–Whitney *U* test between two groups of four measurements (Supplementary Figure S5A). Before performing the statistical tests, we applied a minor normalization to the data from the ETS1 + RUNX1 experiment to ensure that the data (i.e. the fluorescence intensities that represent ETS1 binding levels) were comparable between the ETS1 + RUNX1 and the ETS1-only experiments. Briefly, we normalized the ETS1 + RUNX1 data so that, for the DNA sequences without RUNX1 binding sites (Supplementary Table S2B), the best fit line between the ETS1-only and the ETS1 + RUNX1 data sets was the diagonal (Supplementary Figure S6).

We also removed from further consideration all genomic sequences with ETS1 binding signals lower than the 75th percentile of negative control probes in the ETS1-only experiment (Supplementary Table S2C), as we expect these sequences to be bound mostly non-specifically. Importantly, we chose the $75\%$ rather than $100\%$ cutoff in order to include putative ETS1 sites with very low affinity that might benefit significantly from cooperative binding with RUNX1. After these steps, we obtained 2,161 sequences from the ETS1–RUNX1 experiment, with 976 (45%) of sequences showing cooperative binding (Supplementary Table S2A).

The approach described above assessed the effect of RUNX1, as the ‘cooperator TF’, on ETS1-DNA binding. We also performed the reverse test, which we refer to as the ‘RUNX1-ETS1’ experiment, by considering ETS1 as the cooperator (at 25 nM concentration) and testing its effect on DNA-binding by RUNX1, at 2 nM concentration. The results of RUNX1–ETS1 analyses are shown in Supplementary Table S3, Supplementary Figures S7–S11 and discussed in Supplementary Discussion.

### Identification of ETS1–ETS1 cooperative versus independent binding events

To identify sites cooperatively bound by ETS1, for each genomic region of interest that contained two ETS1 sites (e.g. Figure 1E), we also included in our DNA library three probes generated by mutating one or both of the binding sites, using mutations that are likely to render the binding non-specific (Supplementary Figure S3). We refer to these probes as M1, M2 and M3 (Figure 1F). We performed ETS1 binding measurements (Supplementary Table S4, Supplementary Figure S12) for the mutated sequences, as well the wild-type (WT) sequence, each represented in three replicate spots, at a protein concentration of 10 nM.

Next, for each WT probe (i.e. genomic sequence containing two neighboring ETS1 sites), we asked whether its protein binding level was larger than the sum of the binding levels at probes containing the individual sites (i.e. M1 and M2), adjusting for the level of non-specific binding at these probes (i.e. M3). Thus, to test for cooperative ETS1 binding at the sites in the WT probe we formed the null hypothesis *H*_0_: μ_*WT*_ − μ_*M*3_ = μ_*M*1_ − μ_*M*3_ + μ_*M*2_ − μ_*M*3_ and the alternative hypothesis *H*_1_: μ_*WT*_ − μ_*M*3_ > μ_*M*1_ − μ_*M*3_ + μ_*M*2_ − μ_*M*3_, where μ denotes the mean over replicate measurements (Figure 1H). Finally, we simplified our hypotheses by adding μ_*M*3_ to both sides of the equations above, to obtain *H*_0_: μ_*WT*_ = μ_*M*1_ + μ_*M*2_ − μ_*M*3_ and *H*_1_: μ_*WT*_ > μ_*M*1_ + μ_*M*2_ − μ_*M*3_. Wild-type probes for which we rejected the null hypothesis were considered ‘cooperative’ (Supplementary Table S4); we used the most stringent p-value cutoff (0.0003) for the one-sided Mann–Whitney *U* test applied to the three replicate measurements for WT versus the 27 measurements for M1 + M2–M3 (Supplementary Figure S5B). Wild-type probes with *P* >0.1 were considered ‘independent’, also known as ‘additive’ (38) since the binding level at the two-site probes was equal to the sum of the binding levels at the individual sites. In this study, we use the term ‘independent’ binding, for consistency with the ETS1–RUNX1 analyses.

As in previous work (14), and as described above for the ETS1–RUNX1 analyses, we filtered out WT sequences in the negative control range. In addition, to ensure that the introduced mutations indeed abolished the two binding sites, we filtered out all probes for which M3 was outside of the negative control range. In total, we identified 447 cooperative and 874 independent binding events at neighboring ETS1–ETS1 sites (Supplementary Table S4).

### Designing genomic features to distinguish cooperative versus independent TF binding to neighboring sites

To investigate the rules that govern cooperative versus independent binding of TFs to neighboring sites, we trained classification models to distinguish between cooperative and independent binding events, using as training data the genomic sequences labeled as described above, based on cooperative gcPBM data. Feature design is critical for training accurate and interpretable classification models. Here, we designed features based on the genomic sequences themselves, taking into account characteristics important for TF–DNA recognition.

**Figure 2.**
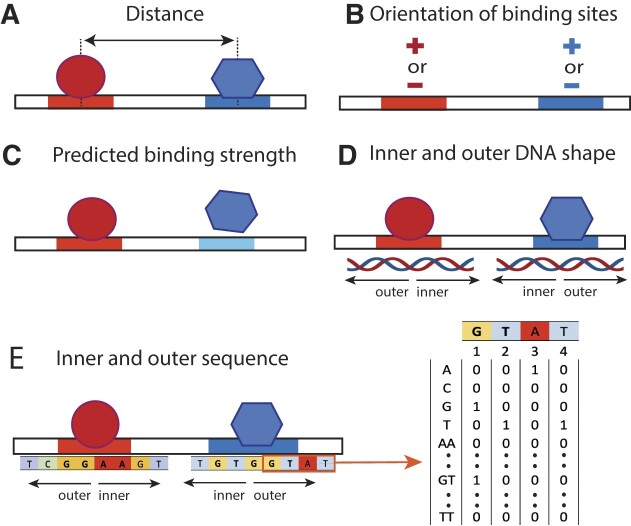
Features used to train classification models. (**A**) Distance between the two sites, (**B**) binding site orientation (defined based on PWM models), (**C**) predicted binding site strengths (using PWM scores), (**D**) positional DNA shape features, (**E**) positional k-mer features, shown for *k* = 1 and 2.

Figure 2 shows the main categories of features used in our analyses: (i) the distance between the two binding sites; (ii) the orientations of the binding sites, which can either be positive (if the site matches the PWM in Figure 1B, E) or negative (if the site matches the reverse complement of the PWM) and (iii) the strengths of the two binding sites, predicted using the PWM models. In addition, we designed features to represent the sequence composition and the DNA shape at and around the binding sites. We computed sequence and shape features taking into account the core binding sites (4 bp for ETS1 and 5 bp for RUNX1, as in (14)), and up to 4 positions in the inner and outer flanks (Figure 2D, E). Shape features (minor groove width, roll, propeller twist and helical twist) were computed using DNAshape (39), and the identities of mono-, di- and trinucleotides were used to compute sequence features, as in (14).

We trained Random Forest classification models using the sklearn.ensemble module in Python, and tuned the parameters n_estimators, max_depth, min_samples_leaf, and min_samples_split using nested 10-fold cross-validation (Supplementary Table S5). Given that the types of features used are very diverse, Random Forest models allowed us to account for nonlinear relationships between our features and the class. To complement the classification models, we also trained regression models to predict the *levels* of cooperative binding between TFs. Details on the implementation and testing of the regression models are available in Supplementary Methods, Supplementary Tables S6–S8, and Supplementary Figures S18–S24 .

## Results

### Cooperative gcPBM data reveals ETS1 cooperative binding to neighboring sites

**Figure 3.**
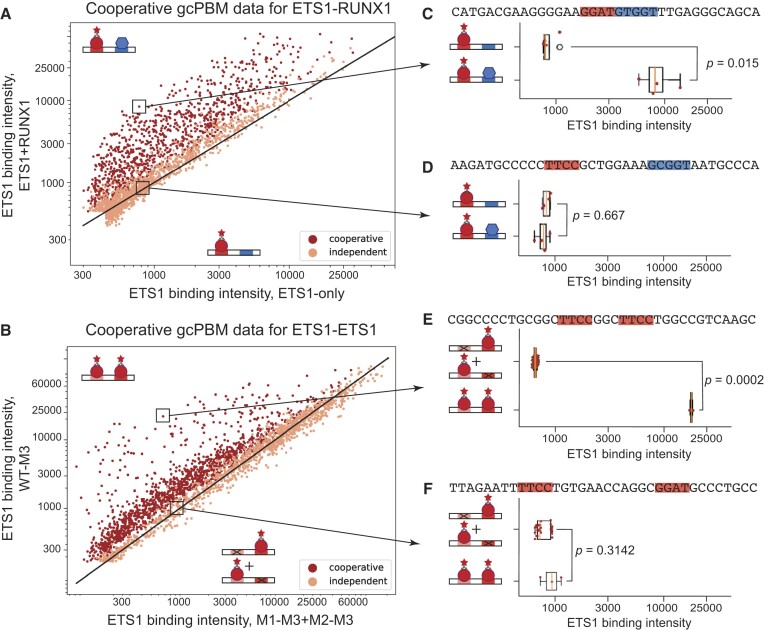
Cooperative versus independent binding revealed by cooperative gcPBM data. (**A**) Comparison between the ETS1 binding intensity in the absence of RUNX1 (x-axis) versus the presence of RUNX1 (y-axis). Each point corresponds to a genomic DNA sequence with neighboring ETS1 and RUNX1 sites. Values shown are medians over four replicate spots. (**B**) Comparison between the ETS1 binding intensity at genomic loci containing two neighboring ETS1 sites (y-axis) versus the sum of binding intensities at the individual sites (x-axis). Each point corresponds to a genomic DNA sequence with two neighboring ETS1 sites. Values shown are medians over three replicate spots. (C, D) Examples of probes bound cooperatively (**C**) and independently (**D**) from the ETS1–RUNX1 experiment. (**E, F**) Similar to (C, D), but for the ETS1–ETS1 experiment. Boxplots show median signals, with boxes extending to the 25th and 75th percentiles. Whiskers extend to the largest/smaller values no further than 1.5 times the inter-quartile range. Points show the individual binding intensity measurements.

As expected, our data showed that ETS1 binds DNA cooperatively with itself, as well as with RUNX1 (Figure 3). The patterns of cooperativity, however, and the features that distinguish cooperative from independent binding events were different for the ETS1–ETS1 versus the ETS1–RUNX1 systems, as described in the sections below. Even the fractions of cooperative binding events identified in the two systems were different: ∼45% for ETS1–RUNX1 and ∼34% for ETS1–ETS1, suggesting that ETS1–ETS1 cooperativity might be more restrictive compared to ETS1–RUNX1, i.e. it might depend more on specific features of the neighboring binding sites, such as distance and orientation.

The genomic sequences included in our DNA libraries were selected in an unbiased manner, based simply on high-throughput *in vivo* binding data for ETS1 and RUNX1, and without biasing the library toward known sites of cooperative interaction between these proteins. Nevertheless, as described below, our data and results are in great agreement with previous, low-throughput studies on ETS1–ETS1 and ETS1–RUNX1 interactions, and they help strengthen and refine previous observations on the cooperative binding of the two proteins to their neighboring sites across the human genome.

### Features encoded in the DNA are highly predictive of ETS1 cooperative binding

**Figure 4.**
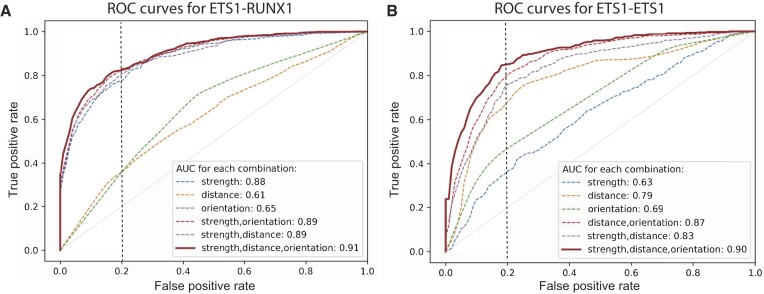
Random Forest classification models can accurately distinguish between cooperative and independent binding events for (**A**) the ETS1–RUNX1 system, and (**B**) the ETS1–ETS1 system. For each combination of features, the false positive and true positive rates were computed by averaging over the 10 folds of a cross-validation test. Dotted vertical lines show the 0.2 false positive rate.

To investigate the determinants of cooperative vs. independent DNA-binding by ETS1 and RUNX1, we trained classification models on our cooperative gcPBM data using features derived from the genomic sequences containing the binding site pairs (Figure 2). Using the area under receiver-operating characteristic curve (AUC), we found that genomic features are highly predictive of cooperative binding (Figure 4), although different features are important in the ETS1–RUNX1 compared to the ETS1–ETS1 systems.

### Binding site strength is highly predictive of ETS1–RUNX1 cooperative DNA binding

**Figure 5.**
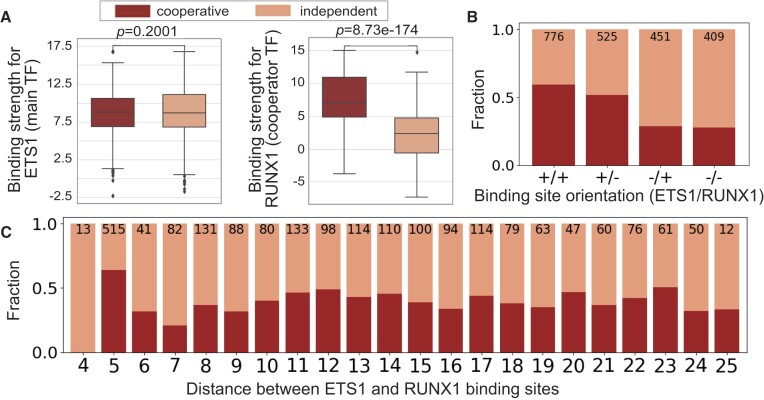
Features used to train cooperative binding models on the ETS1–RUNX1 data. (**A**) Binding site score distributions for sequences with cooperative versus independent binding. Y-axes show PWM scores. *P*-values are according to Mann–Whitney *U* tests. (**B**, **C**) Fractions of probes bound cooperatively versus independently, for different orientations and distances between the ETS1 and RUNX1 binding sites. Numbers above the barplots show the total number of sequences in each category.

For the ETS1–RUNX1 system, the most predictive feature type was the binding site strength (AUC: 0.88, Figure 4A), and in particular the binding strength of the cooperator TF (Supplementary Figure S13A), which is significantly different between cooperatively and independently bound regions (*P* < 10^−173^, Figure 5A). In other words, our results indicate that at genomic regions containing one ETS1 and one RUNX1 site in close proximity, the higher the affinity of the RUNX1 site (and thus the stronger the RUNX1 binding), the more likely we are to observe that ETS1 benefits from cooperative binding with RUNX1. A similar trend was observed in the RUNX1–ETS1 experiment, where the strength of the cooperator’s binding site (in this case, ETS1) was the most predictive feature for distinguishing cooperative from independent binding events (Supplementary Figures S13B, S14, S15). The orientations of the binding sites and the distance between them had only minor contributions to model accuracy (Figure 4A, Supplementary Figure S14), consistent with the fact that we observed cooperative binding at all orientations and almost all distances tested (Figure 5B, C; Supplementary Figure S15B, C).

This result suggests that the cooperative ETS1–RUNX1 binding to DNA is likely driven by protein-protein interactions through domains that are flexible enough to act at a wide range of distances and relative orientations of the binding sites. Our data argues against cooperativity through the DNA, e.g. by allosteric DNA changes, as a dominating mechanism of ETS1–RUNX1 cooperativity, given the large variety of binding site sequences and configurations that can be bound cooperatively by the two proteins.

The hypotheses above are fully consistent with literature. Studies of select ETS1–RUNX1 binding site pairs (28) showed that the cooperativity between the two TFs was retained even when nicks were introduced between their binding sites, thus arguing against a potential role for effects through the DNA. Instead, ETS1 and RUNX1 were shown to enhance each other’s binding to DNA primarily through direct interactions between their auto-inhibitory domains (27–30). The DNA-binding activity of ETS1 is negatively regulated by inhibitory regions that pack against the DNA-binding domain, with the N-terminal region of the inhibitory module unfolding upon ETS1-DNA binding (22,40–42). RUNX1 enhances ETS1–DNA binding by interacting directly with ETS1, disrupting the packing of the auto-inhibitory module and thus countering the auto-inhibition; this can increase the DNA-binding affinity of ETS1 by up to ∼30-fold (28). Furthermore, the crystal structure of the ETS1–RUNX1–DNA ternary complex (30) supports the hypothesis of a flexible linker region in the RUNX1 protein that connects its DNA-binding and its ETS1-interaction domains, consistent with our data showing cooperative binding at a wide range of binding site distances and orientations (Figure 5).

### The role of binding site distance and orientation in ETS1–RUNX1 cooperative DNA binding

The distance between neighboring binding sites can be a strong determinant of cooperative TF–TF interactions (5,43). For the ETS1–RUNX1 system, however, we found that the distance feature does not contribute significantly to the accuracy of our predictive model (Figure 4A), suggesting only a small effect on cooperativity (at least in the range of distances tested). This finding is in agreement with previous, small-scale studies, and generalizes the results of those studies by significantly expanding the number of sequences tested.

Wotton *et al.* (26), Sun *et al.* (44) and Goetz *et al.* (28) reported cooperative binding between ETS1 and RUNX1 when their sites were at distances *d* = 5, 7–9, 12–14, 16, 19, 27 and 38; for most of these distances only a single pair of sites, and thus a single orientation, was tested. Our data revealed cooperative binding between ETS1 and RUNX1 at all distances *d* = 5–25, and all orientations, thus strengthening the point that the cooperativity between these proteins is largely insensitive to the distance between their DNA-binding sites. Nevertheless, the fraction of cooperatively-bound regions was different at different distances, varying from 63.9% at *d* = 5 to 20.7% at *d* = 7 (Figure 5).

The distance *d* = 5 is clearly the most preferred for ETS1–RUNX1 cooperativity: it showed the largest fraction of cooperative binding events (63.9%, or 329/515) and it contains the most probes in our library (515 out of 2161; Figure 5), which means that it is the most common distance between ETS1 and RUNX1 sites in the genomic regions co-bound *in vivo*. Cooperativity between ETS1 and RUNX1 at *d* = 5, and specifically in the +/+ orientation (where 81.8%, or 266/325 regions were labelled as cooperative; Supplementary Table S6), has been widely investigated in molecular, structural and bioinformatic studies (26–28,30,44,45). This is the configuration (i.e. the spacing and orientation) between the known cooperative ETS1–RUNX1 sites in the TCRα and TCRβ enhancers, and it showed the largest cooperativity (∼33-fold) in a synthetic sequence tested by Goetz *et al.* (28). Furthermore, Hollenhorst *et al.* (45) reported that a composite motif corresponding to this configuration was enriched in ETS1-bound genomic regions near genes important for T-cell activation, regions also bound by RUNX1 *in vivo*. No other ETS1–RUNX1 configurations were enriched in the ChIP-seq data, so the authors speculated that this spacing and orientation may also be important for transcriptional activation. Our data, however, suggests that this particular configuration is highly preferred even for the DNA-binding itself.

**Figure 6.**
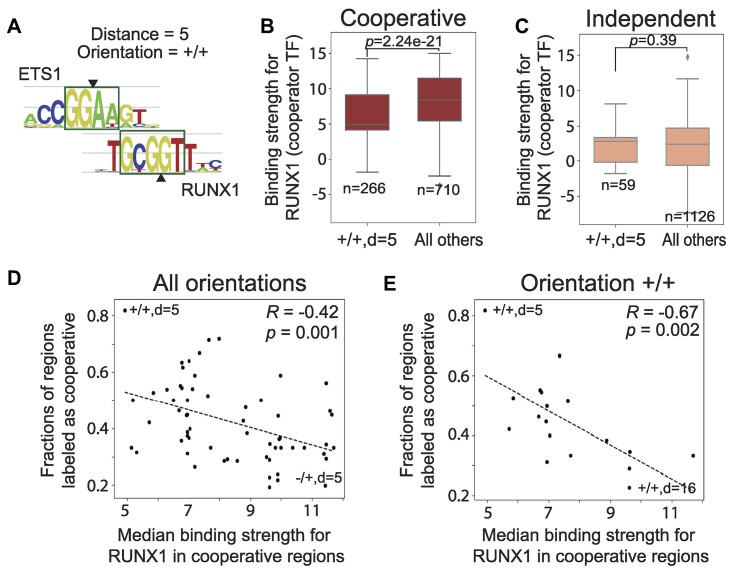
(**A**) Alignment of ETS1 and RUNX1 motifs in the +/+, *d* = 5 configuration. (**B**) Cooperative regions in the +/+, *d* = 5 orientation have lower affinity RUNX1 binding sites compared to cooperative regions in all other configurations. (**C**) In independent regions, the RUNX1 binding affinity is not different. (**D**) Correlation between RUNX1 binding strength and fraction of genomic regions with cooperative ETS1–RUNX1 binding. Each point represents one configuration, i.e. distance and orientation. The x-axis shows the median RUNX1 binding strength over all cooperative regions with a particular configuration. The y-axis shows how ‘preferred’ each configuration is for cooperative binding, assessed by the fraction of cooperative binding events observed at regions with that configuration. Only configurations with five or more regions in our DNA library are shown. (**E**) Same as panel D, but showing only configurations with the +/+ orientation.

Interestingly, when comparing the ETS1–RUNX1 sites in this configuration versus all other configurations present in our DNA library, we found that cooperative regions with +/+ orientation and *d* = 5 (Figure 6A) have lower RUNX1 binding affinity than cooperative binding regions with other configurations (Figure 6B); no significant difference was observed for the independent regions (Figure 6C), nor for the ETS1 binding strength (Supplementary Figure S16A). Considering all configurations tested in our study, we found a significant negative correlation between the RUNX1 binding strength of cooperatively bound sites and the fraction of cooperative sites observed in that configuration (Figure 6D); the trend was even stronger when focusing on the +/+ orientation (Figure 6E), and it was not significant for independently bound sites (Supplementary Figure S16B, C). In other words, our results suggest that in order for ETS1 to benefit from cooperativity with RUNX1, a strong affinity RUNX1 site is generally required; however, at certain ‘preferred’ configurations (identified here as configurations at which we see large fractions of cooperative binding events), lower affinity RUNX1 binding sites can also lead to cooperative binding. This observation is consistent with the fact that the distance and orientation features help improve our model of cooperative versus independent ETS1–RUNX1 binding, although the RUNX1 binding strength feature is the most important for model accuracy (Figure 4A).

Overall, our results are consistent with small-scale studies showing an apparent insensitivity of ETS1 and RUNX1 to the orientation and spacing between their binding sites (22,26–28). In addition, our high-throughput data and analyses reveal a more nuanced view of ETS1–RUNX1 cooperativity (Supplementary Figure S16D, E), where the precise configuration of the neighboring sites does play a role in the cooperative interaction, although the binding strength of the cooperator (here, RUNX1) is the main determinant.

### Binding site distance and orientation are predictive of ETS1–ETS1 cooperative binding

For the ETS1–ETS1 system, we found that cooperative binding depends mainly on the distance between the two sites (AUC 0.79; Figure 4B). Nevertheless, adding strength and orientation features significantly improved the model performance: AUC = 0.87 for distance+orientation and AUC = 0.83 for distance+strength. When all three types of features were used, model performance was further improved, reaching an AUC of 0.90. This trend is clearly different from what we observed in the ETS1–RUNX1 system, where binding strength was the dominant feature and distance and orientation had only minor contributions (Figure 4B), suggesting a different mechanism of cooperativity for ETS1–ETS1 versus ETS1–RUNX1, as discussed below.

**Figure 7.**
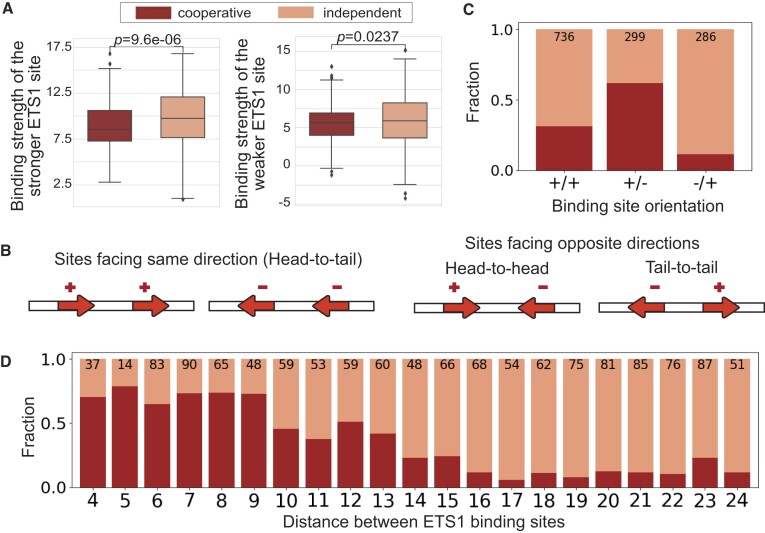
Features used to train cooperative binding models on the ETS1–ETS1 data. (**A**) Binding site score distributions for sequences with cooperative vs. independent binding. Y-axes show PWM scores. P-values are according to Mann–Whitney *U* tests. Separate plots are shown for the stronger site (left) and the weaker site (right). (**B**) Relative orientations for neighboring ETS1 binding sites. Arrow tips mark the ‘head’ of ETS1 binding sites. (**C, D**) Fractions of probes bound cooperatively vs. independently, for different orientations and distances between the neighboring ETS1 binding sites. Numbers above the barplots show the total numbers of sequences in each category.

Closer examination of the cooperative versus independent sites for the ETS1–ETS1 system (Figure 7D) revealed a clear pattern: as the distance between the two neighboring ETS1 sites increases, the fraction of cooperative binding events observed at that distance decreases. For distances d=4-9, more than $65\%$ of binding events were cooperative, for *d* = 10–15 we found between $23\%$ and $51\%$ of neighboring sites to be bound cooperatively, while at larger distances the fraction of cooperative events was typically $< 12\%$. These observations suggests that a physical interaction between structured, rigid domains of the two ETS1 molecules is a likely mechanism for achieving cooperative DNA-binding, as opposed to an interaction through flexible regions that can extend to a wide range of distances, which was the case for ETS1–RUNX1.

The orientation between neighboring sites is also an important determinant of ETS1–ETS1 cooperative binding (AUC = 0.69; Figure 4B), with the +/– orientation showing the largest fraction of cooperative binding events ($62\%$, Figure 7C), followed by +/+ with $31\%$ and –/+ with $12\%$. This trend suggests, yet again, that the cooperative ETS1–ETS1 interactions are likely mediated through direct contacts between specific regions of the two ETS1 molecules, which occur preferentially when the binding sites are in the +/- orientation, i.e. at GGAA-gap-TTCC sequences.

Indeed, co-crystal structures of ETS1 molecules bound to neighboring DNA sites confirm the cooperativity mechanism suggested by our data. At the human stromelysin-1 promoter, two molecules of ETS1 are known to bind neighboring sites in the +/– orientation (GGAAgcacTTCC) at adjacent major grooves (15,31). Structural data revealed direct contacts (hydrogen bonds and van der Waals) between Gly333 and Pro334 of one ETS1 molecule and Asn380 and Lys379 of the other ETS1 (15,25). In addition, mutation analyses showed that cooperative binding at this promoter was abolished when residues Gly333, Pro334 and Asn380 were mutated (15,25). These data indicate a mechanism of cooperative binding facilitated by DNA-mediated homodimerization of ETS1, with the two ETS1 molecules overcoming DNA-binding auto-inhibition through direct interactions between well-structured domains.

Contrary to the ETS1–RUNX1 system, in the case of ETS1–ETS1 interactions we found that the binding strengths of the two neighboring sites are not strong predictors of cooperative vs. independent binding (AUC = 0.63; Figure 4B). To include binding site strength features in our models, for each pair of neighboring ETS1 sites we identified the stronger site and the weaker site, and we used their PWM-based binding scores as features. Each feature by itself had even lower prediction accuracy than the two features together (AUC = 0.61; Supplementary Figure S17). A closer examination of the two features for regions bound cooperatively versus independently by ETS1 showed no significant difference in binding strength for the weaker ETS1 site and a moderate difference for the stronger ETS1 site (Figure 7A). Overall, these results are consistent with our general expectation that cooperativity occurs more often at weaker TF binding sites. However, in the ETS1–ETS1 system, binding site distance and orientation are the most predictive features of cooperative vs. independent binding, consistent with previous biochemical and structural studies (15,25,31).

### Using DNA shape and sequence features to distinguish cooperative versus independent binding

For both the ETS1–RUNX1 and the ETS1–ETS1 systems, we found that features reflecting the binding strengths of the two neighboring sites improve the performance of our classification models. Mechanistically, the strength of TF–DNA interactions is determined by direct (base) readout and indirect (shape) readout (46). Thus, we next asked whether the improvement in classification accuracy when adding binding strength information was due to specific DNA sequence and/or DNA shape features of the binding sites and their flanking regions. To answer this question we trained Random Forest classification models using DNA sequence and shape features, as described in Materials and Methods.

**Figure 8.**
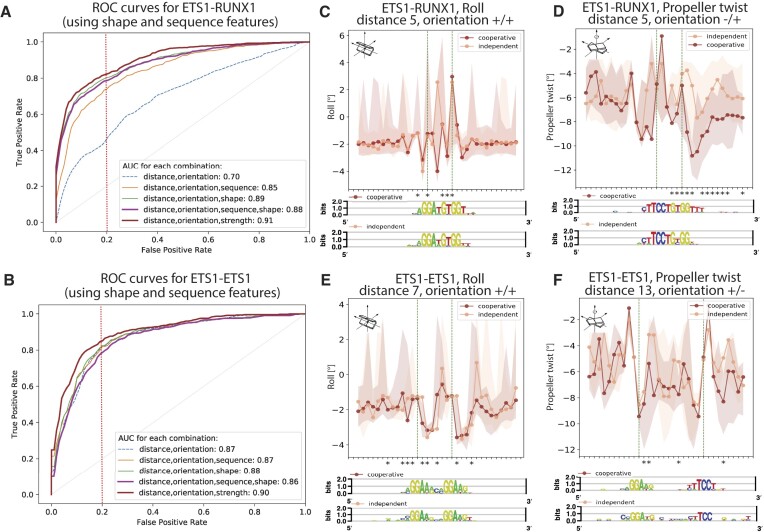
DNA sequence and shape features are predictive of cooperative vs. independent binding. (**A, B**) ROC curves for Random Forest models using DNA shape and sequence features, compared to models using distance, orientation, and binding site strength. (**C–F**) Examples of DNA shape and sequence differences between sites with cooperative versus independent binding, for probes with various distances between the binding site centers. Stars (*) mark positions with significant differences in shape features between cooperative and independent sites (*P* < 0.05, one-sided Mann–Whitney *U* test). Sequence motifs for the cooperative and independent binding events are shown.

Our predictions revealed that DNA sequence and shape features are indeed predictive of cooperative vs. independent binding, although they cannot reach the prediction accuracy of binding strength features (Figure 8A, B). This could be due to the fact that additional structural information is needed to fully describe sites with cooperative binding events, including potential information on the structural deformations induced by the two proteins, which is not currently available but may be intrinsically captured by the binding strength features.

Focusing on the configurations with the most data in our study, we observed that, as expected, the DNA shape and sequence features do differ around sites with cooperative vs. independent binding, for both the ETS1–RUNX1 (Figure 8C, D) and the ETS1–ETS1 (Figure 8E, F) systems. Furthermore, the predictive power of DNA shape features, as observed here, is consistent with a prior study of cooperative TF binding (47).

### Neural network models can predict the level of cooperative TF binding

When two TFs bind cooperatively to neighboring DNA sites, the level of cooperativity can differ depending on the specific properties of the sites. To ask whether computational models can accurately predict the level of cooperative TF binding, we trained regression models on the cooperative gcPBM data to predict the difference between the level of binding when the sites are bound cooperatively and the level expected for independent binding events (see Supplementary Methods for details). Using the correlation between real and predicted binding differences to evaluate our regression models, we found that neural networks performed best. Using one-hot encoding to represent the sequences of the binding sites and their flanks, and adding the predicted binding strengths as input features, neural networks predicted cooperativity levels with a squared Pearson correlation coefficient (*R*^2^) of 0.60 in the ETS1–ETS1 system and 0.72 in the ETS1–RUNX1 system. Further details and discussion of the regression models are available in the Supplementary Material.

## Discussion

Cooperativity is an inherent feature of eukaryotic TF binding to DNA (5), and can occur through diverse mechanisms that do not necessarily depend on direct interactions between TFs (43,48). Even when TF–TF contacts are involved, the DNA can play an important role in facilitating the interaction, by bringing the TFs in closer proximity or by altering the proteins’ conformations to favor specific TF–TF contacts (5). Pairs of cooperating TFs can be identified using large-scale *in vitro* assays, such as CAP-SELEX (12), or from *in vivo* chromatin immunoprecipitation data combined with deep learning modeling (49). Still, even when we know that two TFs can bind DNA cooperatively, the precise determinants and potential mechanisms of cooperativity oftentimes remain unknown. Here, we developed a strategy to study cooperative binding of TFs to DNA using quantitative, high-throughput *in vitro* assays and computational modeling, and applied our approach to the ETS1–RUNX1 and ETS1–ETS1 systems.

Our cooperative binding data showed a surprisingly large number of binding site configurations where cooperativity can occur between ETS1 and RUNX1 (Figure 5, Supplementary Figure S15), while previous studies focused primarily on the configuration described by the composite ETS1–RUNX1 motif GGATGYGGY (26,30,45,45), which is abundant in regulatory regions important for ETS1’s role in T-cells (45). This configuration (+/+ orientation, d=5) is also the most prevalent and most preferred according to our study (Figure 5B, C), but our data shows that many other configurations can lead to cooperative binding. An in depth literature search revealed that, indeed, ETS1 and RUNX1 bind cooperatively at other distances and orientations to regulate their target genes. For example, Sun *et al.* (44) and Goetz *et al.* (28) found neighboring ETS1–RUNX1 binding sites at a distance *d* = 9 and in the +/+ orientation in the Moloney murine leukemia virus enhancer, and mutations in either of the ETS1 or RUNX1 binding sites were found to alter viral disease specificity (50), pointing to the physiological relevance of these additional cooperative configurations. Similarly to ETS1–RUNX1, in the ETS1–ETS1 system we found cooperative interactions at several binding site configurations (Figure 7B–D). Only one configuration (orientation = +/–, *d* = 8) was captured by HT-SELEX data in the form of a composite motif (11), and this is also the configuration present in the stromelysin-1 promoter and analyzed extensively in structural studies (15). Thus, our study extends beyond previous findings in both the ETS1–RUNX1 and ETS1–ETS1 systems, pointing to additional configurations at which the TFs can bind cooperatively.

A caveat of our study, though, is that the cooperative interactions that we observed *in vitro* may not always happen *in vivo*. For example, the two interacting TFs could have other protein partners in the cell, which might prevent the direct interaction between ETS1 and RUNX1 even when their sites are in configurations favorable for cooperativity. In addition, it is possible that some binding site configurations are enriched *in vivo* not because they facilitate the cooperative binding of TFs to their neighboring sites, but because of other *in vivo* factors such as interactions with co-activators or co-repressors. When such interactors are known or hypothesized, they can be added to the gcPBM assays to directly test their influence on TF binding and cooperativity. Overall, we believe that combining the results of *in vitro* and *in vivo* high-throughput assays is the ideal approach for understanding combinatorial gene regulation driven by TF proteins.

One of the known mechanisms for ETS1 cooperativity is through direct physical contacts with its inhibitory regions, which counteracts the auto-inhibition (51). Interestingly, in the two systems studied here, different regions of the cooperator TFs are used to contact the ETS1 inhibitory region, influencing the DNA configurations at which cooperativity occurs. In the ETS1–RUNX1 system, the inhibitory module of ETS1 is displaced by the ETS-interacting domain of RUNX1 (27–30). These interactions are flexible and occur at various distances and orientations (26,44). Meanwhile, the ETS1–ETS1 binding is based on more rigid interactions, involving alpha helices from the inhibitory regions of each ETS1 molecule (15,25). Our cooperative binding data and models are fully consistent with the different mechanisms by which ETS1 auto-inhibition is countered in the two systems: cooperative ETS1–RUNX1 binding depends mainly on the strength of the RUNX1 binding site, with many binding site configurations being amenable to cooperativity, while ETS1–ETS1 cooperativity occurs primarily in the +/- orientation and is largely restricted to sites in very close proximity. These modes of cooperativity through physical TF–TF interactions also explain why our data did now reveal any clear trends dependent on the DNA helical turn, which would have been expected if cooperativity were achieved through DNA-mediated mechanisms (43). In addition, the fact that the ETS–RUNX1 cooperative gcPBM DNA library used in our study contained probes where one or both of the binding sites were mutated allowed us to exclude tethered DNA-binding as a major determinant of the increased binding of one TF in the presence of the cooperator TF (Supplementary Figure S25).

Our approach can be applied to study cooperative TF binding in any system where the TF(s) of interest can be purified as recombinant proteins. In addition, although in this study we leveraged the PBM technology to obtain high-throughput binding measurements and infer cooperative vs. independent binding events, our approach can also be used with sequencing-based assays such as Spec-seq or HT-SELEX/SELEX-seq (11,12,52,53), as long as quantitative and comparable measurements of TF binding can be collected under conditions where the TF binds alone vs. in cooperation with its partner.

In previous studies, SELEX-based methods (CAP-SELEX/HT-SELEX (11,12,47,54)) using randomized DNA libraries have proven very useful in identifying which TFs can bind cooperatively, as well as their most preferred configurations for cooperative binding. However, even for these preferred configurations, TF binding may not always be cooperative, as shown by our data (Figures 5 and 7). In addition, when cooperativity happens at a wide range of distances and at different binding site orientations, as observed in our study, capturing all cooperative binding events by SELEX-based enrichment methods is not feasible. Furthermore, CAP-SELEX/HT-SELEX assays are typically performed using randomized libraries with millions of DNA sequences. While the large diversity of a randomized library can be viewed as an advantage (55), the downside of using such libraries is that the resulting binding data is dominated by high affinity sites, since SELEX methods are based on exponential enrichment of strongly bound sites. Medium and low affinity sites are poorly represented, although they are exactly the types of sites that benefit most from cooperative interactions (7,12). Smaller libraries of genomic DNA sequences, such as the ones used here, help alleviate this shortcoming of randomized libraries and are able to capture TF binding at medium and low affinity sites and identify independent and cooperative binding events at such sites.

As demonstrated by our analyses, binding measurements for just a few thousand genomic sequences with different binding site configurations are sufficient to train accurate classification and regression models of TF cooperativity, and to obtain new insights into the cooperative binding mechanisms of the TFs of interest. In this work, both mechanisms studied involved protein-protein contacts (albeit through different TF domains, and having different degrees of flexibility). In future studies, it will be interesting to investigate TF pairs for which cooperativity is mediated by the DNA through allosteric interactions (5,43). In such systems, we expect that our modeling approach using local DNA sequence and shape features will be very well suited to identify the determinants of cooperative TF binding.

As an alternative to *in vitro* approaches, the recent deep learning-based approach by Avsec *et al.* (49) (BPNet) showed that cooperative interactions between TFs can also be inferred from *in vivo* binding data, by using interpretable deep learning models to determine whether the presence of a cofactor’s binding motif is important for predicting binding of a TF *in vivo*. However, BPNet models capture a mix of effects coming from direct cooperativity between the two TFs (which is the focus of our study) and indirect cooperativity through the competition with nucleosomes (48), which is also widespread in the human genome. The approach we described here, combining *in vitro* binding measurements for a few thousand sites with classification and regression modeling, aims to complement approaches based on *in vivo* data and to provide a deeper understanding of the features that are important for cooperative versus independent binding of TFs to their neighboring DNA sites, across a wide range of affinities and binding site configurations.

## Supplementary Material

gkad872_Supplemental_Files

## Data Availability

The cooperative gcPBM data underlying this article are available in the Gene Expression Omnibus, and can be accessed under accession number GSE171735. The source code for all analyses presented in this manuscript is available in Zenodo at https://doi.org/10.5281/zenodo.8209036.

## References

[1] Ezer D. , Zabet N.R., Adryan B. Homotypic clusters of transcription factor binding sites: a model system for understanding the physical mechanics of gene expression. Comput. Struct. Biotechnol. J. 2014; 10:63–69. 25349675 10.1016/j.csbj.2014.07.005 PMC4204428

[2] Gotea V. , Visel A., Westlund J.M., Nobrega M.A., Pennacchio L.A., Ovcharenko I. Homotypic clusters of transcription factor binding sites are a key component of human promoters and enhancers. Genome Res. 2010; 20:565–577. 20363979 10.1101/gr.104471.109 PMC2860159

[3] Lifanov A.P. , Makeev V.J., Nazina A.G., Papatsenko D.A. Homotypic regulatory clusters in Drosophila. Genome Res. 2003; 13:579–588. 12670999 10.1101/gr.668403 PMC430164

[4] Lu R. , Rogan P.K. Transcription factor binding site clusters identify target genes with similar tissue-wide expression and buffer against mutations. F1000Res. 2018; 7:1933. 31001412 10.12688/f1000research.17363.1 PMC6464064

[5] Morgunova E. , Taipale J. Structural perspective of cooperative transcription factor binding. Curr. Opin. Struct. Biol. 2017; 47:1–8. 28349863 10.1016/j.sbi.2017.03.006

[6] Siggers T. , Gordan R. Protein-DNA binding: complexities and multi-protein codes. Nucleic Acids Res. 2014; 42:2099–2111. 24243859 10.1093/nar/gkt1112 PMC3936734

[7] Datta V. , Siddharthan R., Krishna S. Detection of cooperatively bound transcription factor pairs using ChIP-seq peak intensities and expectation maximization. PLoS One. 2018; 13:e0199771. 30016330 10.1371/journal.pone.0199771 PMC6049898

[8] Shively C. , Liu J., Chen X., Loell K., Mitra R. Homotypic cooperativity and collective binding are determinants of bHLH specificity and function. Proc. Natl. Acad. Sci. U.S.A. 2019; 116:16143–16152. 31341088 10.1073/pnas.1818015116 PMC6689977

[9] Slattery M. , Riley T., Liu P., Abe N., Gomez-Alcala P., Dror I., Zhou T., Rohs R., Honig B., Bussemaker H.J. et al. Cofactor binding evokes latent differences in DNA binding specificity between Hox proteins. Cell. 2011; 147:1270–1282. 22153072 10.1016/j.cell.2011.10.053 PMC3319069

[10] Tugrul M. , Paixao T., Barton N.H., Tkacik G. Dynamics of transcription factor binding site evolution. PLoS Genet. 2015; 11:e1005639. 26545200 10.1371/journal.pgen.1005639 PMC4636380

[11] Jolma A. , Yan J., Whitington T., Toivonen J., Nitta K.R., Rastas P., Morgunova E., Enge M., Taipale M., Wei G. et al. DNA-binding specificities of human transcription factors. Cell. 2013; 152:327–339. 23332764 10.1016/j.cell.2012.12.009

[12] Jolma A. , Yin Y., Nitta K.R., Dave K., Popov A., Taipale M., Enge M., Kivioja T., Morgunova E., Taipale J. DNA-dependent formation of transcription factor pairs alters their binding specificity. Nature. 2015; 527:384–388. 26550823 10.1038/nature15518

[13] Gordan R. , Shen N., Dror I., Zhou T., Horton J., Rohs R., Bulyk M.L. Genomic regions flanking E-box binding sites influence DNA binding specificity of bHLH transcription factors through DNA shape. Cell Rep. 2013; 3:1093–1104. 23562153 10.1016/j.celrep.2013.03.014 PMC3640701

[14] Shen N. , Zhao J., Schipper J.L., Zhang Y., Bepler T., Leehr D., Bradley J., Horton J., Lapp H., Gordan R. Divergence in DNA specificity among paralogous transcription factors contributes to their differential in vivo binding. Cell Syst. 2018; 6:470–483. 29605182 10.1016/j.cels.2018.02.009 PMC6008103

[15] Lamber E.P. , Vanhille L., Textor L.C., Kachalova G.S., Sieweke M.H., Wilmanns M. Regulation of the transcription factor Ets-1 by DNA-mediated homo-dimerization. EMBO J. 2008; 27:2006–2017. 18566588 10.1038/emboj.2008.117 PMC2486274

[16] Fitzsimmons D. , Hodsdon W., Wheat W., Maira S.M., Wasylyk B., Hagman J. Pax-5 (BSAP) recruits Ets proto-oncogene family proteins to form functional ternary complexes on a B-cell-specific promoter. Genes Dev. 1996; 10:2198–2211. 8804314 10.1101/gad.10.17.2198

[17] Sieweke M.H. , Tekotte H., Frampton J., Graf T. MafB represses erythroid genes and differentiation through direct interaction with c-Ets-1. Leukemia. 1997; 11:486–488. 9209434

[18] Sieweke M.H. , Tekotte H., Jarosch U., Graf T. Cooperative interaction of ets-1 with USF-1 required for HIV-1 enhancer activity in T cells. EMBO J. 1998; 17:1728–1739. 9501094 10.1093/emboj/17.6.1728 PMC1170520

[19] Bradford A.P. , Wasylyk C., Wasylyk B., Gutierrez-Hartmann A. Interaction of Ets-1 and the POU-homeodomain protein GHF-1/Pit-1 reconstitutes pituitary-specific gene expression. Mol. Cell Biol. 1997; 17:1065–1074. 9032233 10.1128/mcb.17.3.1065 PMC231831

[20] Gégonne A. , Bosselut R., Bailly R.A., Ghysdael J. Synergistic activation of the HTLV1 LTR Ets-responsive region by transcription factors Ets1 and Sp1. EMBO J. 1993; 12:1169–1178. 8458329 10.1002/j.1460-2075.1993.tb05758.x PMC413320

[21] Wasylyk B. , Wasylyk C., Flores P., Begue A., Leprince D., Stehelin D. The c-ets proto-oncogenes encode transcription factors that cooperate with c-Fos and c-Jun for transcriptional activation. Nature. 1990; 346:191–193. 2114554 10.1038/346191a0

[22] Garvie C.W. , Pufall M.A., Graves B.J., Wolberger C. Structural analysis of the autoinhibition of Ets-1 and its role in protein partnerships. J. Biol. Chem. 2002; 277:45529–45536. 12221090 10.1074/jbc.M206327200

[23] Choy W.W. , Datta D., Geiger C.A., Birrane G., Grant M.A. Crystallization and preliminary X-ray analysis of a complex of the FOXO1 and Ets1 DNA-binding domains and DNA. Acta Crystallogr. F. Struct. Biol. Commun. 2014; 70:44–48. 24419615 10.1107/S2053230X13024795 PMC3943110

[24] Baillat D. , Begue A., Stehelin D., Aumercier M. ETS-1 transcription factor binds cooperatively to the palindromic head to head ETS-binding sites of the stromelysin-1 promoter by counteracting autoinhibition. J. Biol. Chem. 2002; 277:29386–29398. 12034715 10.1074/jbc.M200088200

[25] Babayeva N.D. , Wilder P., Shiina M., Mino K., Desler M., Ogata K., Rizzino A., Tahirov T.H. Structural basis of Ets1 cooperative binding to palindromic sequences on stromelysin-1 promoter DNA. Cell Cycle. 2010; 9:3054–3062. 20686355 10.4161/cc.9.15.12257 PMC2928650

[26] Wotton D. , Ghysdael J., Wang S., Speck N.A., Owen M.J. Cooperative binding of Ets-1 and core binding factor to DNA. Mol. Cell Biol. 1994; 14:840–850. 8264651 10.1128/mcb.14.1.840-850.1994 PMC358432

[27] Kim W.Y. , Sieweke M., Ogawa E., Wee H.J., Englmeier U., Graf T., Ito Y. Mutual activation of Ets-1 and AML1 DNA binding by direct interaction of their autoinhibitory domains. EMBO J. 1999; 18:1609–1620. 10075931 10.1093/emboj/18.6.1609 PMC1171248

[28] Goetz T.L. , Gu T.L., Speck N.A., Graves B.J. Auto-inhibition of Ets-1 is counteracted by DNA binding cooperativity with core-binding factor alpha2. Mol. Cell Biol. 2000; 20:81–90. 10594011 10.1128/mcb.20.1.81-90.2000 PMC85055

[29] Gu T.L. , Goetz T.L., Graves B.J., Speck N.A. Auto-inhibition and partner proteins, core-binding factor beta (CBFbeta) and Ets-1, modulate DNA binding by CBFalpha2 (AML1). Mol. Cell Biol. 2000; 20:91–103. 10594012 10.1128/mcb.20.1.91-103.2000 PMC85059

[30] Shrivastava T. , Mino K., Babayeva N.D., Baranovskaya O.I., Rizzino A., Tahirov T.H. Structural basis of Ets1 activation by Runx1. Leukemia. 2014; 28:2040–2048. 24646888 10.1038/leu.2014.111 PMC4169772

[31] Shiina M. , Hamada K., Inoue-Bungo T., Shimamura M., Uchiyama A., Baba S., Sato K., Yamamoto M., Ogata K. A novel allosteric mechanism on protein-DNA interactions underlying the phosphorylation-dependent regulation of Ets1 target gene expressions. J. Mol. Biol. 2015; 427:1655–1669. 25083921 10.1016/j.jmb.2014.07.020

[32] Moore J.E. , Purcaro M.J., Pratt H.E., Epstein C.B., Shoresh N., Adrian J., Kawli T., Davis C.A., Dobin A., Kaul R. et al. Expanded encyclopaedias of DNA elements in the human and mouse genomes. Nature. 2020; 583:699–710. 32728249 10.1038/s41586-020-2493-4 PMC7410828

[33] Liu T. , Ortiz J.A., Taing L., Meyer C.A., Lee B., Zhang Y., Shin H., Wong S.S., Ma J., Lei Y. et al. Cistrome: an integrative platform for transcriptional regulation studies. Genome Biol. 2011; 12:R83. 21859476 10.1186/gb-2011-12-8-r83 PMC3245621

[34] Berger M.F. , Bulyk M.L. Universal protein-binding microarrays for the comprehensive characterization of the DNA-binding specificities of transcription factors. Nat. Protoc. 2009; 4:393–411. 19265799 10.1038/nprot.2008.195 PMC2908410

[35] Zhang Y. , Ho T., Buchler N., Gordan R. Competition for DNA binding between paralogous transcription factors determines their genomic occupancy and regulatory functions. Genome Res. 2021; 31:1216–1229. 33975875 10.1101/gr.275145.120 PMC8256859

[36] Penvose A. , Keenan J.L., Bray D., Ramlall V., Siggers T. Comprehensive study of nuclear receptor DNA binding provides a revised framework for understanding receptor specificity. Nat. Commun. 2019; 10:2514. 31175293 10.1038/s41467-019-10264-3 PMC6555819

[37] Afek A. , Shi H., Rangadurai A., Sahay H., Senitzki A., Xhani S., Fang M., Salinas R., Mielko Z., Pufall M.A. et al. DNA mismatches reveal conformational penalties in protein-DNA recognition. Nature. 2020; 587:291–296. 33087930 10.1038/s41586-020-2843-2 PMC7666076

[38] Liu J. , Shively C.A., Mitra R.D. Quantitative analysis of transcription factor binding and expression using calling cards reporter arrays. Nucleic Acids Res. 2020; 48:e50. 32133534 10.1093/nar/gkaa141 PMC7229839

[39] Zhou T. , Yang L., Lu Y., Dror I., Dantas Machado A.C., Ghane T., Di Felice R., Rohs R. DNAshape: a method for the high-throughput prediction of DNA structural features on a genomic scale. Nucleic Acids Res. 2013; 41:56–62. 10.1093/nar/gkt437 PMC3692085 23703209

[40] Petersen J.M. , Skalicky J.J., Donaldson L.W., McIntosh L.P., Alber T., Graves B.J. Modulation of transcription factor Ets-1 DNA binding: DNA-induced unfolding of an alpha helix. Science. 1995; 269:1866–1869. 7569926 10.1126/science.7569926

[41] Jonsen M.D. , Petersen J.M., Xu Q.P., Graves B.J. Characterization of the cooperative function of inhibitory sequences in Ets-1. Mol. Cell Biol. 1996; 16:2065–2073. 8628272 10.1128/mcb.16.5.2065 PMC231193

[42] Skalicky J.J. , Donaldson L.W., Petersen J.M., Graves B.J., McIntosh L.P. Structural coupling of the inhibitory regions flanking the ETS domain of murine Ets-1. Protein Sci. 1996; 5:296–309. 8745408 10.1002/pro.5560050214 PMC2143348

[43] Kim S. , Brostromer E., Xing D., Jin J., Chong S., Ge H., Wang S., Gu C., Yang L., Gao Y.Q. et al. Probing allostery through DNA. Science. 2013; 339:816–819. 23413354 10.1126/science.1229223 PMC3586787

[44] Sun W. , Graves B.J., Speck N.A. Transactivation of the Moloney murine leukemia virus and T-cell receptor beta-chain enhancers by cbf and ets requires intact binding sites for both proteins. J. Virol. 1995; 69:4941–4949. 7609063 10.1128/jvi.69.8.4941-4949.1995 PMC189309

[45] Hollenhorst P.C. , Chandler K.J., Poulsen R.L., Johnson W.E., Speck N.A., Graves B.J. DNA specificity determinants associate with distinct transcription factor functions. PLoS Genet. 2009; 5:e1000778. 20019798 10.1371/journal.pgen.1000778 PMC2787013

[46] Rohs R. , Jin X., West S.M., Joshi R., Honig B., Mann R.S. Origins of specificity in protein-DNA recognition. Annu. Rev. Biochem. 2010; 79:233–269. 20334529 10.1146/annurev-biochem-060408-091030 PMC3285485

[47] Ibarra I.L. , Hollmann N.M., Klaus B., Augsten S., Velten B., Hennig J., Zaugg J.B. Mechanistic insights into transcription factor cooperativity and its impact on protein-phenotype interactions. Nat. Commun. 2020; 11:124. 31913281 10.1038/s41467-019-13888-7 PMC6949242

[48] Wasson T. , Hartemink A.J. An ensemble model of competitive multi-factor binding of the genome. Genome Res. 2009; 19:2101–2112. 19720867 10.1101/gr.093450.109 PMC2775586

[49] Avsec Z. , Weilert M., Shrikumar A., Krueger S., Alexandari A., Dalal K., Fropf R., McAnany C., Gagneur J., Kundaje A. et al. Base-resolution models of transcription-factor binding reveal soft motif syntax. Nat. Genet. 2021; 53:354–366. 33603233 10.1038/s41588-021-00782-6 PMC8812996

[50] Speck N.A. , Renjifo B., Golemis E., Fredrickson T.N., Hartley J.W., Hopkins N. Mutation of the core or adjacent LVb elements of the Moloney murine leukemia virus enhancer alters disease specificity. Genes Dev. 1990; 4:233–242. 2338244 10.1101/gad.4.2.233

[51] Graves B.J. , Cowley D.O., Goetz T.L., Petersen J.M., Jonsen M.D., Gillespie M.E. Autoinhibition as a transcriptional regulatory mechanism. Cold Spring Harb Symp. Quant. Biol. 1998; 63:621–629. 10384327 10.1101/sqb.1998.63.621

[52] Stormo G.D. , Zuo Z., Chang Y.K. Spec-seq: determining protein—DNA-binding specificity by sequencing. Brief. Funct. Genom. 2014; 14:30–38. 10.1093/bfgp/elu043 PMC4366588 25362070

[53] Zhang L. , Martini G., Rube H., Kribelbauer J., Rastogi C., FitzPatrick V., Houtman J., Bussemaker H., Pufall M. SelexGLM differentiates androgen and glucocorticoid receptor DNA-binding preference over an extended binding site. Genome Res. 2018; 28:111–121. 29196557 10.1101/gr.222844.117 PMC5749176

[54] Jolma A. , Kivioja T., Toivonen J., Cheng L., Wei G., Enge M., Taipale M., Vaquerizas J.M., Yan J., Sillanpää M.J. et al. Multiplexed massively parallel SELEX for characterization of human transcription factor binding specificities. Genome Res. 2010; 20:861–873. 20378718 10.1101/gr.100552.109 PMC2877582

[55] de Boer C.G. , Taipale J. Hold out the genome: a roadmap to solving the cis-regulatory code. 2023; bioRxiv doi:20 April 2023, preprint: not peer reviewed 10.1101/2023.04.20.537701. 38093018

