# Supplementary material for: High-throughput data and modeling reveal insights into the mechanisms of cooperative DNA-binding by transcription factor proteins
Source: Nucleic Acids Res. 2023 Oct 27;51(21):11600–12. doi: 10.1093/nar/gkad872 (PMC10681739; doi:10.1093/nar/gkad872)
Supplement: gkad872_Supplemental_Files [file gkad872_supplemental_files.zip › Martin_et_al_SupplementaryMaterial.pdf]

Supplementary Material for: High-throughput data and modeling reveal insights into the mechanisms of cooperative DNA-binding by transcription factor proteins

VINCENTIUS MARTIN [1,2], FARICA ZHUANG [1,2], YUNING ZHANG [2,3], KYLE PINHEIRO [1,2], RALUCA GORDÂN [1,2,4] *

[1]Department of Computer Science [2]Center for Genomic & Computational Biology [3]Program in Computational Biology & Bioinformatics [4]Department of Biostatistics & Bioinformatics, Duke University, Durham, NC 27708

CONTENTS

1 Supplementary Methods 1

2 Supplementary Discussion 3

3 Supplementary Tables (Table S1) 7

4 Supplementary Figures 8

5 Supplementary References 23

1. SUPPLEMENTARY METHODS

In addition to training classification models to predict whether neighboring TF binding sites are bound cooperatively or independently, our cooperative gcPBM data can also be used to train regression models that reflect the *level* of cooperativity, i.e. how much TF binding at a particular site increases due to a cooperator TF bound nearby. In this section we describe the methods used for our regression analyses, including how regression labels and datasets were defined, how model architectures were determined via hyperparameter searches, and other implementation details. Performance of the regression models is discussed in Supplementary Discussion below.

Defining the regression labels

We define the regression labels for the ETS1-ETS1 and ETS1-RUNX1 systems differently, due to differences in the design of the two experiments. For ETS1-ETS1, the regression label for a sequence was set to $\mu_{WT} - \mu_{M1} - \mu_{M2} + \mu_{M3}$, which aligns with the experimental quantities used to determine the classification labels for ETS1-ETS1 (**Figure 1H**). The ETS1-RUNX1 regression labels were set to the differences between the ETS1 binding levels in the ETS1+RUNX1 and the ETS1-only experiments. Again, this aligns with the experimental quantities used to assign classification labels (**Figure 1D**).

Datasets for training and testing regression models of cooperative binding

ETS1-RUNX1 regression models were trained on the same genomic sequences used for the classification analyses (**Table S2**). For the ETS1-ETS1 system, though, initial analyses using support vector regression (SVR) and random forest regression (RFR) showed that combining genomic sequences labelled as 'cooperative' or 'independent' (**Table S4**) led to poor overall accuracy (**Figure S18**, blue bars). When focusing only on the sequences

*To whom correspondence should be addressed. Email: raluca.gordan@duke.edu

labelled as 'cooperative', the accuracy of both SVR and RFR increased substantially (**Figure S18**, green bars). The hyperparameters tested for SVR and RFR models are available in **Table S7A,B**. These results suggest that ETS1-RUNX1 cooperativity likely extends beyond the sequences labelled as 'cooperative' in our classification analyses (at a stringent p-value cutoff), so that even regions labelled as 'ambiguous' or 'independent' according to our current data can potentially become cooperative under different conditions, such as an even higher concentration of the cooperator TF (here, RUNX1). Thus, although under the experimental conditions tested some differences between ETS1 binding levels in the ETS1-RUNX1 vs. the ETS1-only experiments were not statistically significant (so the corresponding regions were not labelled 'cooperative'), the differences themselves still contained useful information about the features that determine the level of cooperativity. In contrast, in the case of ETS1-ETS1, cooperativity is restricted to a smaller number of specific configurations and binding sites; thus, when we add the 874 'independent' sequences to the 447 'cooperative' ones, the data becomes too convoluted for the SVR and RFR algorithms to learn accurate models. In other words, it is possible that the features that determine whether ETS1 binding is cooperative (or not) at a particular pair of neighboring sites are different from the features that determine the *level* of cooperativity, which is a more continuous characteristic. Based on these results, we used only genomic sequences labelled as 'cooperative' to train regression models of cooperative ETS1-ETS1 binding. In addition, it is interesting to note that, when investigating which features are most important for accurately predicting the level of cooperativity for ETS1-ETS1 and ETS1-RUNX1, we found that binding strength features had the largest contribution in both systems, with distance and orientation features further increasing the accuracy (**Figures S20, S21**). This was not surprising for the ETS1-RUNX1 system, where binding site strength features were also critical for the performance of classification models. But in the ETS1-ETS1 system, these results indicate that while binding site configuration (i.e. the relative orientation and distance between the neighboring sites) is critical for determining whether two ETS1 molecules bind cooperatively, once the configuration is favorable, the amount by which overall binding increases due to ETS1-ETS1 cooperativity depends primarily on the strength of the sites.

Establishing a target R^2

Given that predicting cooperative binding levels in a regression framework is more difficult than simply predicting whether cooperative binding occurs or not, we analyzed our gcPBM data to determine what prediction accuracy we can expect from the regression models. In particular, we aimed to use replicate measurements of cooperative binding to determine an upper bound for the target R^2 between predicted and measured levels of cooperativity. For this purpose, we leveraged the fact that our gcPBM data sets contain replicate measurements for each DNA sequence of interest (as described in detail in Materials and Methods). Thus, for every sequence we took half of the replicates and computed cooperativity levels based on those measurements, and then repeated the procedure for the second half of the replicates. We then computed the R^2 value between these two sets of cooperativity levels. For both the ETS1-ETS1 and the ETS1-RUNX1 datasets, the R^2 was 0.77, so we set this as the target value against which to compare the performance of our regression models.

Neural network models

In addition to SVR and RFR, we also trained neural network regression models on our cooperative gcPBM data, encoding the input DNA sequences using a one-hot encoding that included 1-mers, 2-mers, or 3-mers. Unlike SVR and RFR, the hyperparameter space for convolutional neural networks (CNN) is large, so it is not feasible to do a grid search across all possible values. Instead, we used three increasingly granular grid searches. We first focused on the number of convolutional layers, and performed

hyperparameter grid searches for CNNs of increasing depths (1 to 7 layers; **Table S7C**). We also varied the kernel widths of each layer, holding the number of filters at each layer and the size of the first fully-connected layer constant. Additionally, in the same grid search we did experiments with and without max-pooling, and with and without including binding strengths as input features. For this grid search, the optimizer settings (specified in **Table S7D**) were held constant. The resulting R^2 performances for these different hyperparameter combinations are summarized in **Figures S23** and **S24**.

In the experiments where binding strengths were added as inputs, the two values (one for each of the two binding sites) were concatenated to the flattened convolutional filter values and input to the first fully-connected layer. In the architectures where max-pooling layers were used, they were placed after the second convolutional layer, and then after every subsequent convolutional layer.

For the ETS1-ETS1 system, the highest scoring non-max pooling architecture had 4 layers, 2-mer encoding, independent binding strength values included, and kernel widths at each layer of sizes 11, 11, 7, and 11. For ETS1-RUNX1, there was negligible improvement with more than 5 layers, so the highest scoring 5-layer architecture was selected, which used 1-mer encoding, no max-pooling, independent binding affinity values, and kernel widths at each layer of sizes 7, 3, 3, 11, and 7. A further grid search for each dataset was executed on a more granular range of hyperparameter values (**Tables S7E, S7F**), and then a final grid search, with kernel sizes at each layer held constant, varying training parameters: weight decay, dropout rate, learning rate, and batch size (**Table S7G**). For ETS1-RUNX1, the optimal hyperparameters were: no dropout, 0.001 weight decay, with 0.0005 learning rate, 128 convolution filters per layer, batch size of 64, fully-connected layer with 1024 nodes (**Table S7I**). For ETS1-ETS1, the optimal hyperparameters were: no dropout or weight decay, a learning rate of 0.0005, batch size of 32, 64 convolution filters per layer, and 512 nodes in the first fully-connected layer (**Table S7H**).

Evaluation of regression models

RFR and SVR were evaluated by averaging the mean R^2 values from five separate 5-fold cross validation tests. In this evaluation phase, the five cross-validations used predetermined shuffles (**Table S8A,B**) so that the splits were reproducible and performance on the same splits could be used to also compare results for CNN models. For the CNNs, the best-scoring parameter sets for each of the two datasets were run through five separate 5-fold cross-validations with the same train-test splits used for evaluating RFR and SVR, and the resulting R^2 values were averaged. The train-test splits can be found in **Table S8**.

Implementation details of regression models

Neural networks were implemented using Pytorch [1] and trained using an Adam optimizer [2] with a mean-squared error loss function. Target values were standardized. SLURM was used to parallelize grid searches, so that model selection could be run across many jobs. RFR and SVR models were trained using `RandomForestRegressor` and `SVR` from scikit-learn [3]. Source code for the CNN experiments and RFR/SVR experiments can be found at: https://github.com/vincentiusmartin/Cooperative-gcPBM/regression, in the respective directories: deep_learning and svr_and_rfr.

2. SUPPLEMENTARY DISCUSSION

Considerations for the choice of antibodies used in TF-TF cooperative studies

Measuring TF binding in cooperative gcPBM assays requires antibodies. This is somewhat similar to *in vivo* assays such as ChIP-seq [4], although the antibodies used in PBM assays do not need to be ChIP-grade because our assays are based on imaging as opposed to immunoprecipitation. Similar to other antibody-based assays, we cannot

completely alleviate all concerns related to antibody effects. However, such potential effects can be minimized by choosing antibodies with known epitopes that are not located within protein domains critical for DNA binding or TF-TF interactions.

In the current study, for TF ETS1 we used a monoclonal antibody produced by immunizing animals with a synthetic peptide corresponding to residues surrounding Ser179 of human ETS1 protein (Cell Signaling Technology, catalog number 14069). Ser179 is located in ETS1's activation domain (a.a. 130-243), which is distal to ETS1's DNA-binding and RUNX1-interacting domains (a.a. 280-441; as annotated in UniProt [5] and literature [6]). For RUNX1 we used a polyclonal antibody (Abcam, catalog number ab23980) produced using as immunogen a synthetic peptide corresponding to human RUNX1 a.a. 200-300. This region does not overlap the DNA-binding domain of RUNX1 (a.a. 50-178). There is small region of overlap with the annotated ETS1-interaction domain of RUNX1 (an alpha-helix located at a.a. 190-214), but co-crystal structures of ETS1-RUNX1-DNA complexes show that the RUNX1 region with direct interactions with ETS1 ends at Leu201 of RUNX1 [6]. We note that other antibodies are available for RUNX1, but they were either lower sensitivity (according to manufacturer's data) or did not specify the target epitope due to proprietary data. For these reasons, we decided to use the two antibodies described above in our study.

As a more general approach, in cases where the TF-TF interacting domains are completely unknown, or when the epitope information for all commercially available antibodies is proprietary, a good strategy would be to express the TFs of interest with N-terminal and C-terminal tags, and use anti-tag antibodies. Consistency between binding data obtained from the two tags would indicate that the antibodies are unlikely to affect the TF-DNA or TF-TF interactions.

The effect of ETS1 on RUNX1-DNA binding at neighboring sites

In the main text, we focused primarily on the effects of RUNX1-DNA binding on ETS1 binding at neighboring sites, i.e. the ETS1-RUNX1 experiment. Conversely, one can also ask whether binding of ETS1 to DNA increases the probability that RUNX1 will bind at a neighboring site (i.e. the RUNX1-ETS1 experiment). Other studies have observed cooperativity in one direction but not in the other [7, 8]. However, we argue that whether we observed cooperativity in one direction or the other depends, to a large extent, on the concentrations of the two proteins. To test this hypothesis, we performed an experiment where we measured the influence of ETS1, present at a saturating level, on RUNX1-DNA binding, i.e. RUNX1-ETS1 (**Figures S7**, **S8**, **S9**; complementary to ETS1-RUNX1 **Figure 1C,D** in the main text and **Figures S4**, **S6**). We found 1,449 cooperative and 324 independent binding events (**Table S3**, **Figure S10**), i.e. ~82% of all genomic RUNX1 sites located in close proximity to ETS1 sites benefited from cooperative binding between the two proteins. In comparison, only ~45% of ETS1 sites benefited from cooperative binding with RUNX1 in the ETS1-RUNX1 experiment, i.e. ETS1 binding at these sites increased significantly in the presence of a saturating level of RUNX1.

To understand the reason behind the apparent discrepancy between ETS1-RUNX1 and RUNX1-ETS1 cooperativity, we examined our data in more depth and we found that the RUNX1 binding sites selected for our DNA library (i.e. the RUNX1 sites that are proximal to ETS1 sites) were generally of much lower affinity than RUNX1 sites found generally within ChIP-seq peaks (**Figure S11A**). This trend was not as pronounced in the case of ETS1, i.e. we found many medium and high affinity genomic ETS1 binding sites in close proximity to RUNX1 sites (**Figure S11B**). This observation also explains why the total numbers of binding events that we could confidently call either cooperative or independent differ between the ETS1-RUNX1 and the RUNX1-ETS1 experiments: many of the RUNX1 sites tested were of very low affinity in the RUNX1-only experiment, and practically indistinguishable from negative controls. Overall, these results show that cooperativity is more frequent at lower affinity sites, and that TF binding sites that are potentially cooperatively bound are not among those with

the highest affinity, consistent with previous studies highlighting the importance of cooperativity at weak/medium-affinity sites [9, 10].

Cooperative versus tethered binding of ETS1 and RUNX1 to the DNA

An important consideration for the interpretation of our cooperative gcPBM data is whether the tested proteins truly bind cooperatively to the DNA, versus the alternative scenario in which the TF-TF interactions form off DNA and potentially lead to binding of only one site at a time (i.e. tethered binding). In the case of ETS1 and RUNX1, our data argues against a significant contribution from the latter mode of binding (**Figure S25**), and this is in agreement with prior literature, including the work of Wotton et al. [11] and Sun et al. [12] who showed that binding of both ETS1 and RUNX1 to the DNA is necessary for cooperative binding.

We were able to investigate the possibility of tethered binding using our high-throughput data because our cooperative gcPBM library includes probes where either the ETS1 site or the RUNX1 site (or both) were mutated. If the ETS1-RUNX1 heterodimer formed off DNA and then bound DNA with only one site bound at a time, then we would expect the sum of TF binding levels at ETS1-only probes plus RUNX1-only probes (minus the non-specific level of binding reflected by the probes with both sites mutated) to be very similar to the TF binding level at wild-type probes that contain both motifs (i.e. ETS1+RUNX1 probes). But, as shown in **Figure S25**, this is not the case. In panel A, which shows ETS1 binding levels in the ETS1+RUNX1 experiment, please compare the red (1st) and dark red (3rd) violins. In panel B, which shows RUNX1 binding levels in the RUNX1+ETS1 experiment, please compare the blue (1st) and dark blue (3rd) violins.

As another way to think about the scenario in which the main TF is tethered to the DNA by the cooperator TF, if the ETS1-RUNX1 complex formed off DNA and bound only one site at a time, then in the ETS1-RUNX1 experiment we would expect ETS1 to be tethered to RUNX1-only probes, leading to significant ETS1 binding levels at these probes. But, as shown in **Figure S25A**, the ETS1 binding level at RUNX1-only probes (light blue violin) is very low. Still, it is important to note that we cannot completely exclude any effect, albeit very modest, due to indirect/tethered DNA-binding by ETS1 through RUNX1, as the ETS1 binding level at RUNX1-only probes (light blue violin) is slightly higher than the binding level at probes with both sites mutated (empty violin). Similar results were observed in RUNX1-ETS1 experiment (**Figure S25B**), where the RUNX1 binding level at ETS1-only probes (orange violin) was very low and comparable to the binding level at probes with both sites mutated (empty violin).

Overall, we conclude that even if the ETS1-RUNX1 heterodimer can form off the DNA and lead to a low amount of tethered DNA-binding of one TF through the other, this mechanism would not explain our cooperative gcPBM data.

Evaluation of regression models

As shown in **Figure S22**, CNN models matched the performance of the best SVR and RFR models on the ETS1-ETS1 dataset and out-performed the best RFR and SVR models on the ETS1-RUNX1 dataset, likely due to the increased representational capacity of CNN models and their ability to extract more information from the DNA sequences than the pre-defined features used for SVR and RFR.

The best CNN model for ETS1-ETS1 achieved an R^2 of 0.60, and the best CNN model for ETS-RUNX1 achieved an R^2 of 0.72, compared to the target performance of 0.77 (computed from replicate measurements). One reason for the lower accuracy of the ETS1-ETS1 model compared to the ETS1-RUNX1 model is likely the difference in the amount of data used for training. As mentioned in Supplementary Methods above, the ETS1-ETS1 regression models were trained on a small number of 447 sequences labelled as 'cooperative' (as described in detail in the main text), while the ETS1-RUNX1 models were trained on 2161 sequences. It is also possible that the true (and unknown)

function that describes the level of ETS1-ETS1 cooperative binding is more complex than for ETS1-RUNX1, which would also point to a need for more data in order to improve the accuracy of the ETS1-ETS1 regression model. As discussed in detail in the main text, ETS1-ETS1 cooperativity is restricted to certain configurations of the binding sites, while the ETS1-RUNX1 cooperativity shows a large degree of flexibility, consistent with the protein-protein interactions in the two systems. In the ETS1-RUNX1 system, features based on strength of the binding sites were able to distinguish independent from cooperative binding events with high accuracy (**Figure 4A**), and such features can be captured well by CNN models. Given the restricted cooperativity in the ETS1-ETS1 system, and the rigid protein domains involved in this interaction, it is possible that structural DNA features beyond the ones available at the moment [13] are needed in order to accurately predict the level of cooperativity.

3. SUPPLEMENTARY TABLES (TABLE S1)

Source	ID	Cell line	Target	Analysis
ENCODE	ENCSR588AKU	K562	RUNX1	ETS1-RUNX1
ENCODE	ENCSR000BKQ	K562	ETS1	ETS1-RUNX1, ETS1-ETS1
ENCODE	ENCSR000BPU	A549	ETS1	ETS1-ETS1
ENCODE	ENCSR000BKA	GM12878	ETS1	ETS1-ETS1
ENCODE	ENCSR534VHI	GM23338	ETS1	ETS1-ETS1
ENCODE	ENCSR681WHQ	HepG2	ETS1	ETS1-ETS1
Cistrome	1938	Jurkat	ETS1	ETS1-RUNX1
Cistrome	1955	Jurkat	RUNX1	ETS1-RUNX1
Cistrome	37927	HUVEC	ETS1	ETS1-ETS1
Cistrome	37934	HUVEC	ETS1	ETS1-ETS1
Cistrome	37936	HUVEC	ETS1	ETS1-ETS1
Cistrome	43097	CUTLL1	ETS1	ETS1-ETS1
Cistrome	44090	Jurkat	ETS1	ETS1-ETS1
Cistrome	44092	Jurkat	RUNX1	ETS1-RUNX1
Cistrome	44093	Jurkat	ETS1	ETS1-ETS1
Cistrome	44094	Jurkat	ETS1	ETS1-ETS1
Cistrome	44097	Jurkat	ETS1	ETS1-RUNX1
Cistrome	46068	A549	ETS1	ETS1-ETS1
Cistrome	46278	K562	ETS1	ETS1-RUNX1
Cistrome	49238	DU145	ETS1	ETS1-ETS1
Cistrome	63823	GM23248	ETS1	ETS1-ETS1
Cistrome	63973	K562	RUNX1	ETS1-RUNX1
Cistrome	72623	HUVEC	ETS1	ETS1-ETS1
Cistrome	72627	HUVEC	ETS1	ETS1-ETS1
Cistrome	72628	HUVEC	ETS1	ETS1-ETS1
Cistrome	90553	HEYA8	ETS1	ETS1-ETS1
Cistrome	93394	HUVEC	ETS1	ETS1-ETS1
Cistrome	93396	HUVEC	ETS1	ETS1-ETS1

Table S1. ENCODE [14] and Cistrome [15] ChIP-seq data sets used to identify genomic sequences with neighboring DNA sites for the ETS1 and RUNX1 transcription factors.

4. SUPPLEMENTARY FIGURES

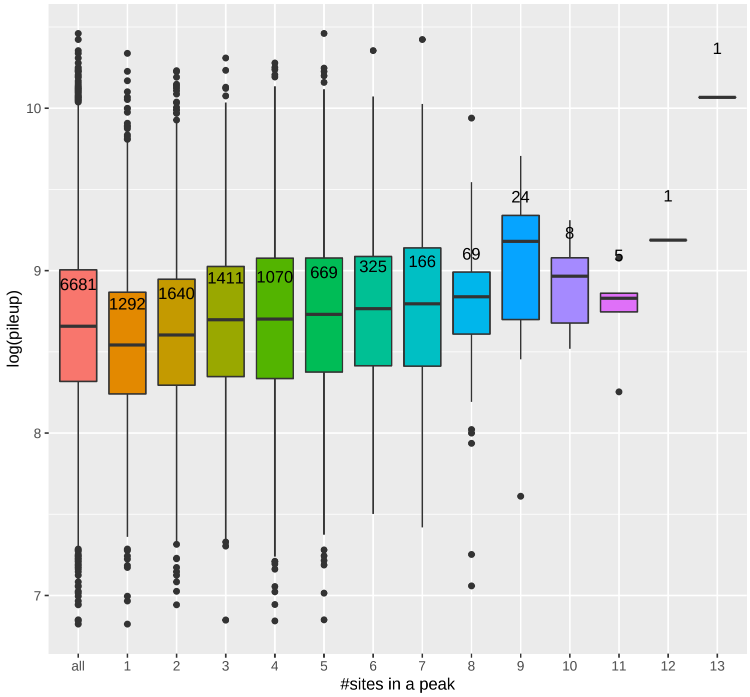

Fig. S1. ChIP-seq peaks with multiple putative ETS1 binding sites have higher ChIP-seq signal, calculated as the natural logarithm of the peak pileup score reported by the MACS software tool (https://github.com/macs3-project/MACS). A representative plot is shown, for the ETS1 ChIP-seq data in K562 cells (ENCODE ID: ENCSR000BKQ). Boxplots show median signals, with boxes extending to the 25th and 75th percentiles. Whiskers extend to the largest/smaller values no further than 1.5 times the inter-quartile range, and the points show the most extreme data points.

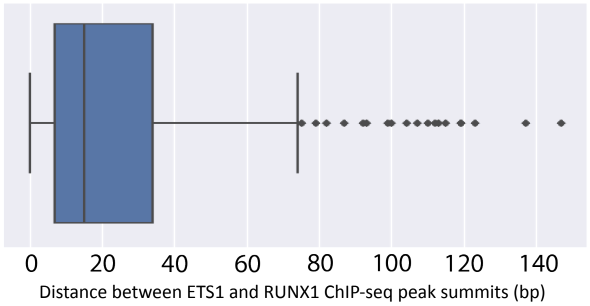

Fig. S2. Distribution of distances between ChIP-seq peak summits for ETS1 and RUNX1. For each RUNX1 ChIP-seq peak summit in K562 cells (ENCODE ID: ENCSR588AKU) we calculated the distance to the nearest ETS1 peak summit (ENCODE ID: ENCSR000BKQ). Boxplot shows the median distance (17 bp), with the box extending to the 25th and 75th percentiles. Whiskers extend to the largest/smaller values no further than 1.5 times the inter-quartile range, and the points show the most extreme data points.

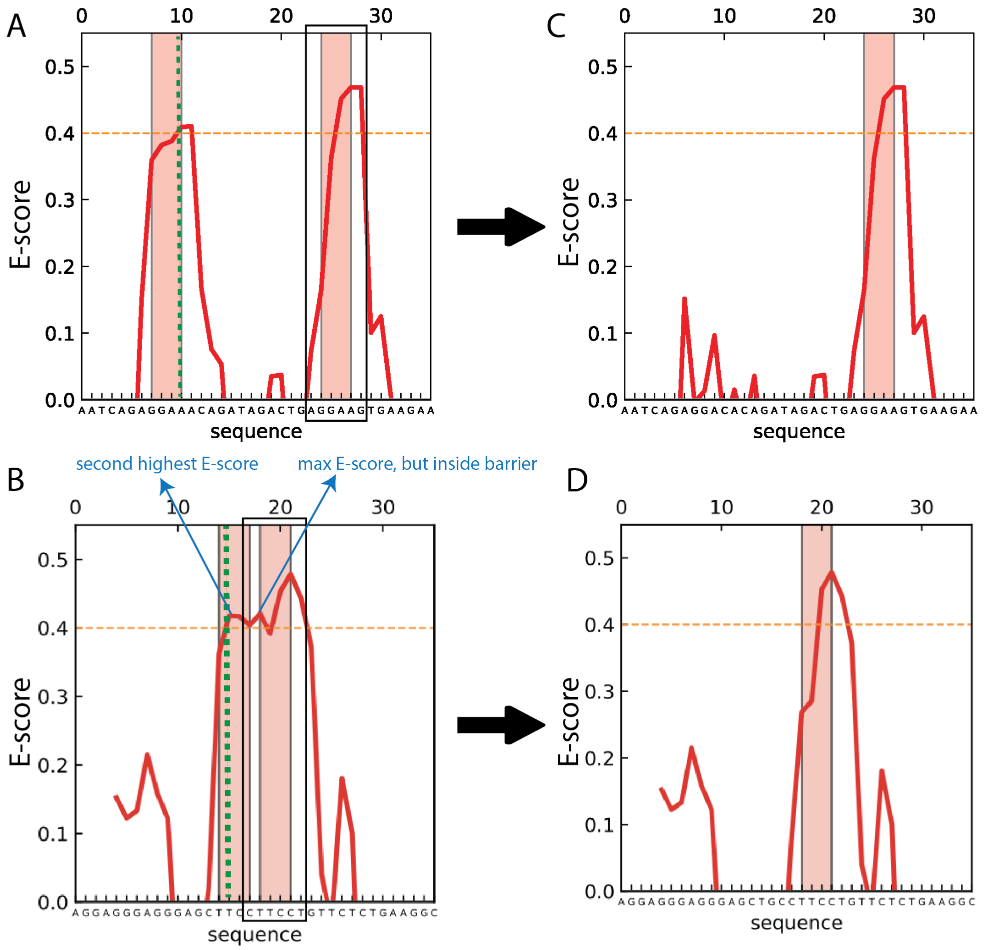

Fig. S3. Designing DNA probes with mutated ETS1 binding sites for the ETS1-ETS1 DNA library. In order to detect cooperative binding of ETS1 to pairs of neighboring sites extracted from the human genome, for each genomic region of interest we generated mutated sequences where one or both of the binding sites were mutated. (A,B) Example genomic regions containing two ETS1 binding sites. The cores of the binding sites (GGAA or TTCC) are highlighted using light red rectangles. The y-axes show the universal PBM E-scores for all 8-mers (red line) covering the genomic sequences shown on the x-axes, focusing on the [0,0.5] range. E-scores above 0.4 typically correspond to specific TF-DNA binding [16, 17]. (C,D) For the genomic regions shown in panels A and B, we illustrate the corresponding mutated probes where the first of the two binding sites was mutated so that none of the 8-mers overlapping the binding core had E-scores above 0.4. To mutate a binding site, we targeted the 8-mer with the largest E-score and we chose the single base-pair mutation that resulted in the largest decrease in E-score. The mutated positions are marked with vertical green dotted lines in panels A and B. To make sure that the neighboring binding site is not affected by the mutation, we defined a ”barrier” region (i.e. positions that could not be mutated) around the binding sites, marked with a black box in panels A and B. If the position with the largest E-score fell within the barrier region (as in the example in panel B), then we chose the next largest E-score position in order to find an appropriate mutation.

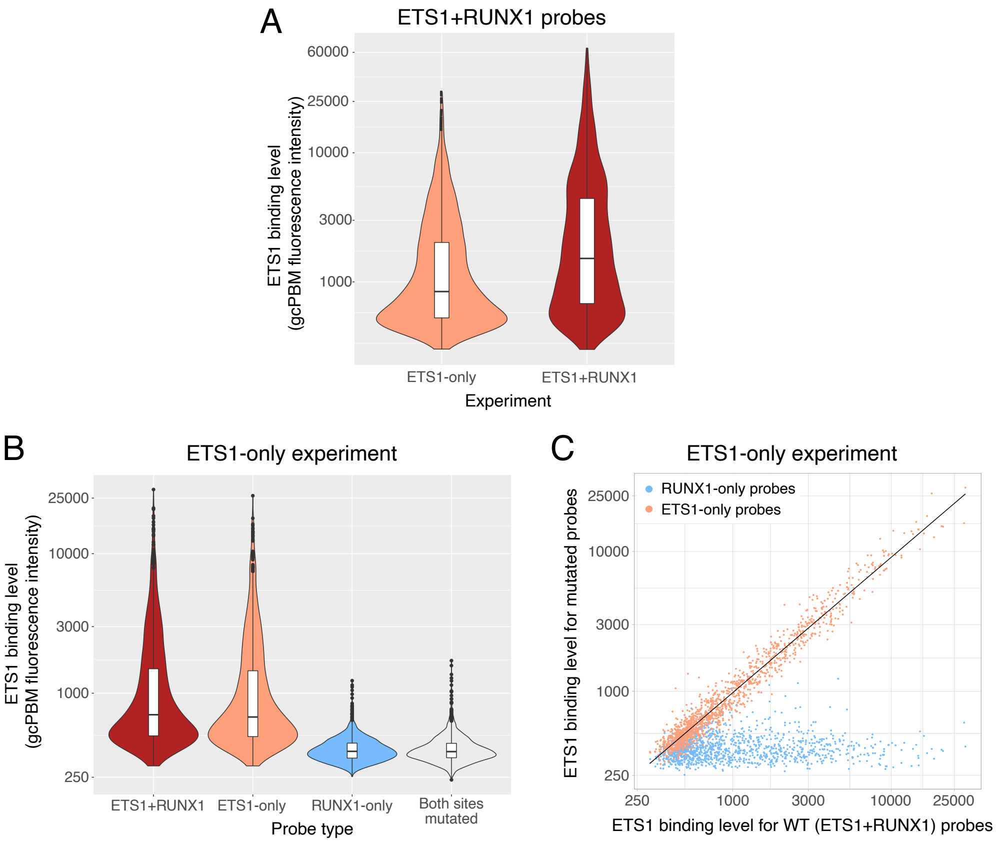

Fig. S4. ETS1 binding. (A) Violin plots showing ETS1 binding levels (gcPBM fluorescence intensities) for DNA sequences containing neighboring ETS1+RUNX1 binding sites, in the ETS1-only (left) and the ETS1+RUNX1 (right) experiments. (B) ETS1 binding levels for probes with both sites mutated (empty violin plot), only the ETS1 site mutated (i.e. RUNX1-only probes, blue violin plot), only the RUNX1 site mutated (i.e. ETS1-only probes, orange violin plot), and wild-type probes (i.e. ETS1+RUNX1 probes, red violin plot). Mann-Whitney U test p-values between the different groups of probes are: p=0.5192 for ETS1+RUNX1 vs. ETS1-only probes, p=0.8505 for RUNX1-only vs. "Both sites mutated" probes, p<2.2e-16 for ETS1+RUNX1 vs. RUNX1-only probes, and p<2.2e-16 for ETS1-only vs. "Both sites mutated" probes. (C) Scatter plot of ETS1 binding levels at WT probes (x-axis) vs. the corresponding probes where either the ETS1 or the RUNX1 site was mutated (y-axis). All data shown in panels B and C are from the ETS1-only experiment, i.e. in the absence of RUNX1 protein.

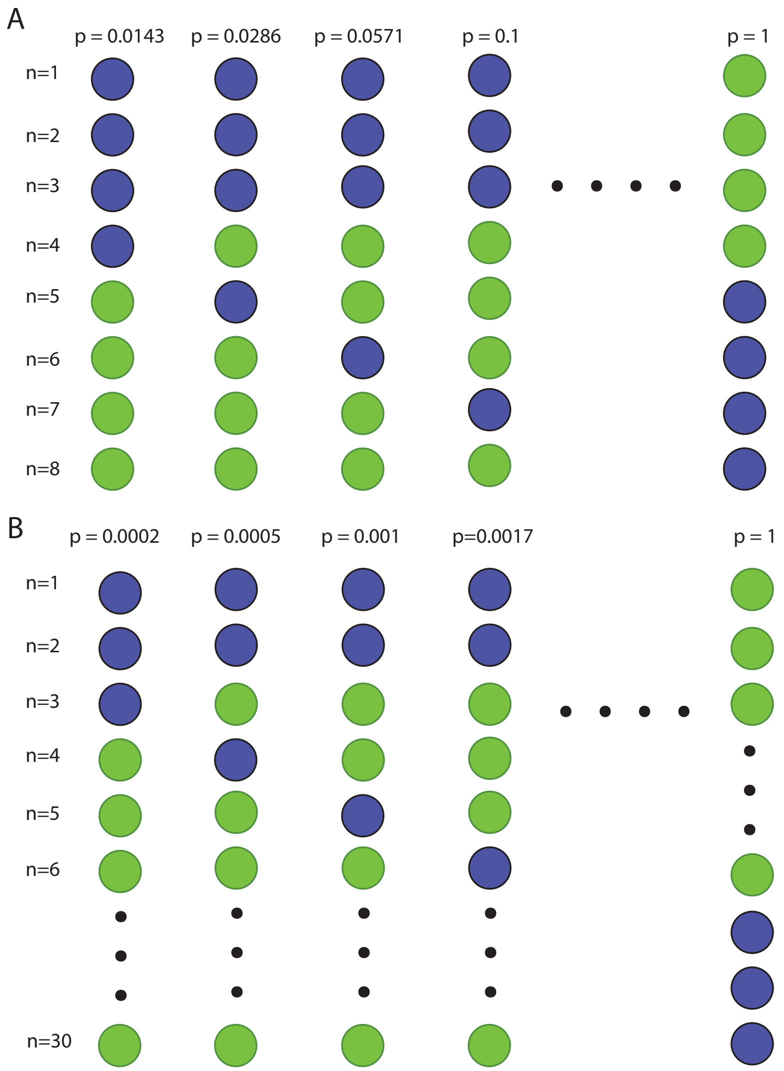

Fig. S5. Possible p-value for a one-sided Mann-Whitney *U* test between two groups of 4 measurements each (A), or a group of 3 measurements compared to a group of 27 measurements (B). The two colors illustrate the two groups. The alternative hypothesis is that the blue group contains higher values than the green group. The data points (measurements) are sorted in decreasing order, with the rank numbers shown on the left. The smallest p-values (left-most) correspond to the situation when the two groups have no overlap, and all values for the blue data points are higher than all values for the green data points.

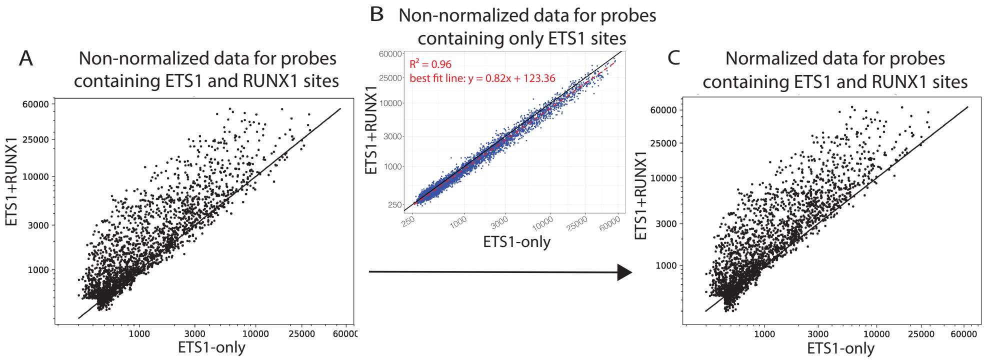

Fig. S6. Normalization of the ETS1 gcPBM data between the ETS1-only (x-axis) and the ETS1+RUNX1 (y-axis) experiments. (A) Comparison between the non-normalized ETS1 binding data from the ETS1-only versus the ETS1+RUNX1 experiments. Binding levels are represented as fluorescence intensities from the two gcPBM experiments. Each data point corresponds to a genomic sequence containing one ETS1 site and one RUNX1 site in close proximity. (B) Similar to panel A, but each point represents a DNA sequence containing one ETS1 site and no RUNX1 site. Black line shows the diagonal. Red dotted line shows the best fit line. (C) Similar to panel A, but the y-axis shows data after normalization, which was performed by applying the function $f(y) = (y - 123.36)/0.82$.

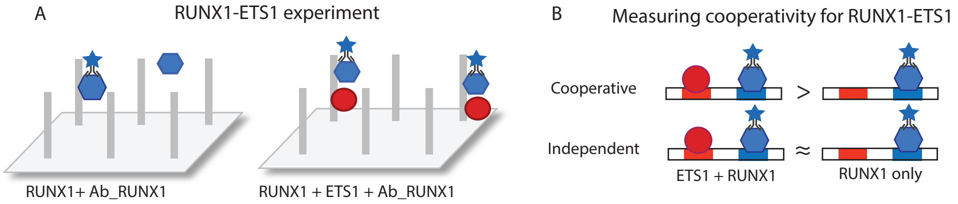

Fig. S7. (A) Cooperative gcPBM experiment designed to detect cooperative binding events by comparing binding of RUNX1 (blue) alone and in the presence of a high concentration of cooperator TF (ETS1, red). Ab_RUNX1: anti-RUNX1 antibody. (B) Binding of RUNX1-ETS1 is considered cooperative when the RUNX1 binding intensity is significantly higher in the presence compared to the absence of ETS1.

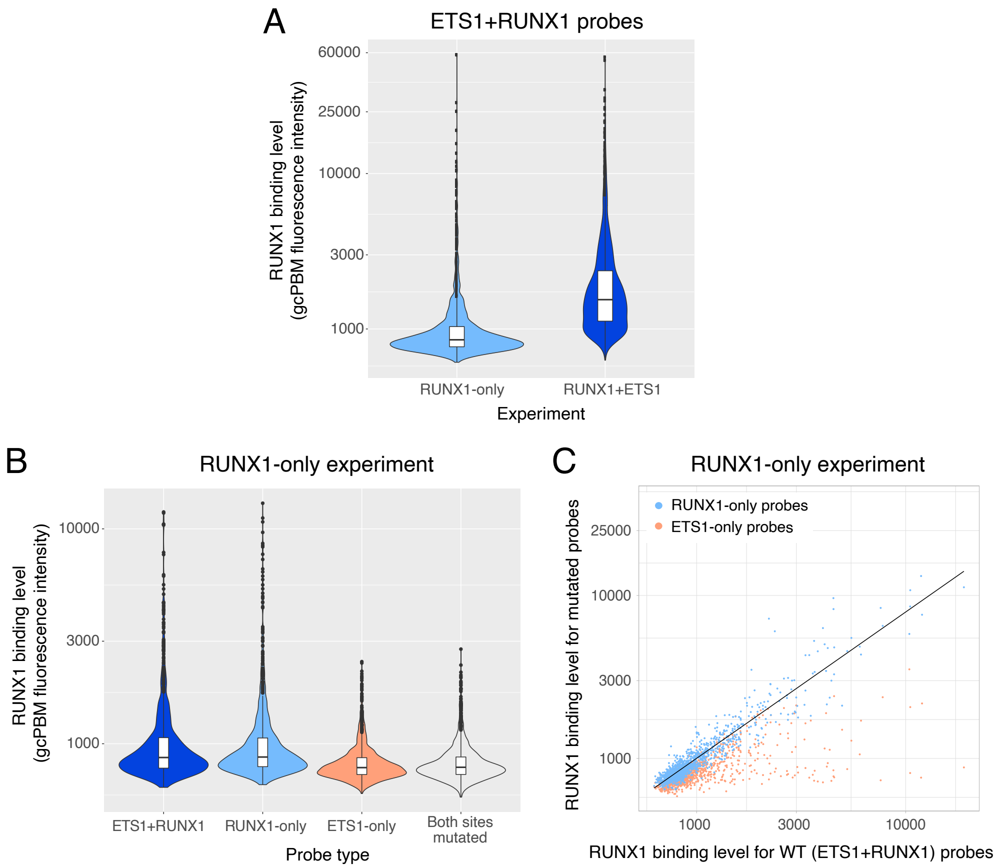

Fig. S8. RUNX1 binding. (A) Violin plots showing RUNX1 binding levels (gcPBM fluorescence intensities) for DNA sequences containing neighboring ETS1+RUNX1 binding sites, in the RUNX1-only (left) and the RUNX1+ETS1 (right) experiments. (B) RUNX1 binding levels for probes with both sites mutated (empty violin plot), only the RUNX1 site mutated (i.e. ETS1-only probes, orange violin plot), only the ETS1 site mutated (i.e. RUNX1-only probes, light blue violin plot), and wild-type probes (i.e. ETS1+RUNX1 probes, dark blue violin plot). Mann-Whitney U test p-values between the different groups of probes are: p=0.2975 for ETS1+RUNX1 vs. RUNX1-only probes, p=0.6453 for ETS1-only vs. "Both sites mutated" probes, p<2.2e-16 for ETS1+RUNX1 vs. ETS1-only probes, and p<2.2e-16 for RUNX1-only vs. "Both sites mutated" probes. (C) Scatter plot of RUNX1 binding levels at WT probes (x-axis) vs. the corresponding probes where either the ETS1 or the RUNX1 site was mutated (y-axis). All data shown in panels B and C are from the RUNX1-only experiment, i.e. in the absence of ETS1 protein. In panels B and C, outliers measurements for the ETS1+RUNX1 probes were omitted in order to enhance the visibility of the low intensity values, especially for the ETS1-only probes.

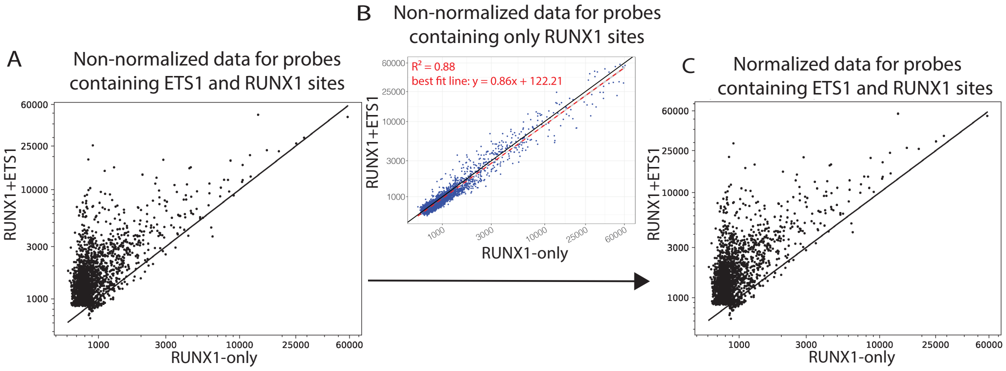

Fig. S9. Normalization of the RUNX1 gcPBM data between the RUNX1-only (x-axis) and the RUNX1+ETS1 (y-axis) experiments. (A) Comparison between the non-normalized RUNX1 binding data from the RUNX1-only versus the RUNX1+ETS1 experiments. Binding levels are represented as fluorescence intensities from the two gcPBM experiments. Each data point corresponds to a genomic sequence contain one ETS1 site and one RUNX1 site in close proximity. (B) Similar to panel A, but each point represents a DNA sequence containing one RUNX1 site and no ETS1 site. Black line shows the diagonal. Red dotted line shows the best fit line. (C) Similar to panel A, but the y-axis shows data after normalization, which was performed by applying the function $f(y) = (y - 122.21)/0.86$.

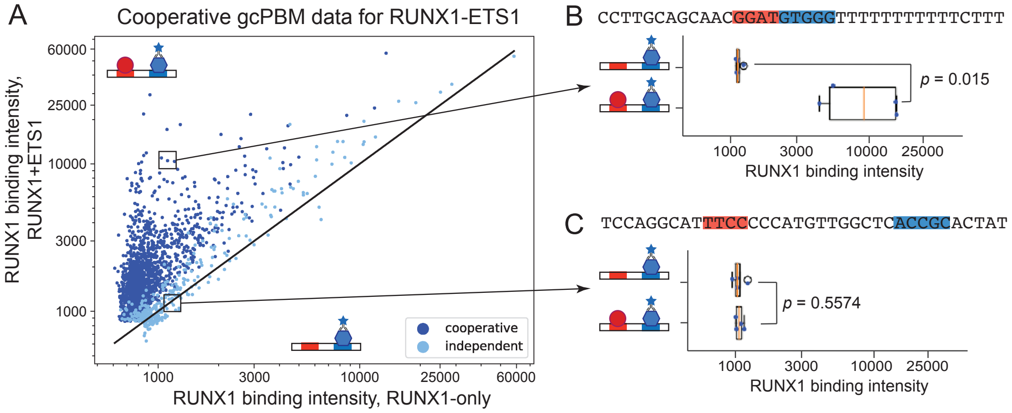

Fig. S10. Cooperative vs. independent binding of RUNX1, revealed by cooperative gcPBM data. (A) Comparison between the RUNX1 binding intensity in the absence of ETS1 (x-axis) versus the presence of ETS1 (y-axis). Each point corresponds to a genomic DNA sequence with neighboring ETS1 and RUNX1 sites. Values shown are medians over four replicate spots. (B,C) Examples of probes bound cooperatively (B) and independently (C) from the RUNX1-ETS1 experiment. Boxplots show median signals, with boxes extending to the 25th and 75th percentiles. Whiskers extend to the largest/smaller values no further than 1.5 times the inter-quartile range. Points show the individual binding intensity measurements. P-values were calculated using a one-sided Mann-Whitney U test.

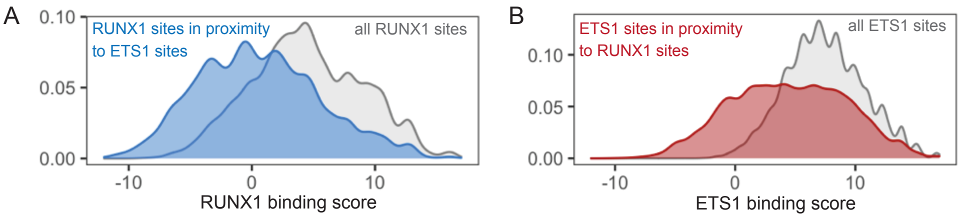

Fig. S11. Distributions of TF binding scores (calculated based on PWM models) for (A) RUNX1 binding sites and (B) ETS1 binding sites in close proximity to ETS1 sites. The binding sites for both ETS1 and RUNX1 are identified from ChIP-seq peaks.

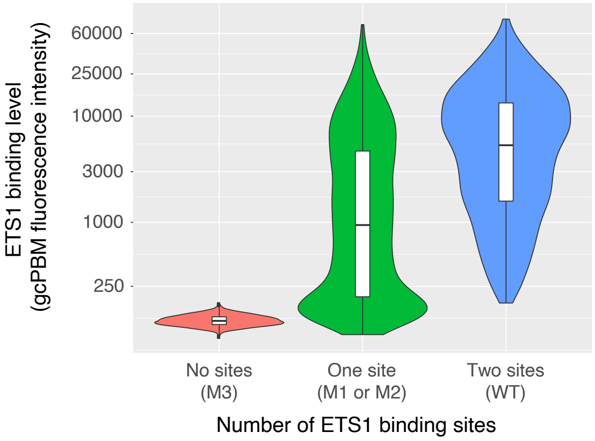

Fig. S12. Violin plots showing ETS1 binding signal (gcPBM fluorescence intensity) for DNA sequences containing two neighboring ETS1 binding sites (right), a single ETS1 binding site (middle) or no ETS1 binding sites (left). See Materials and Methods for details on the design of the DNA probes (WT, M1, M2, M3).

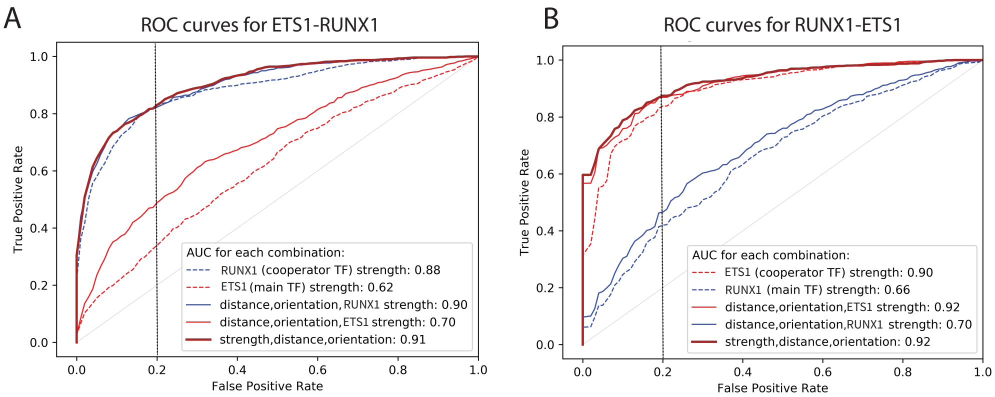

Fig. S13. Performance of Random Forest models trained separately on the binding site strength of either the main TF or the cooperator TF. (A) ETS1-RUNX1 system, where ETS1 is the main TF and RUNX1 is the cooperator. (B) RUNX1-ETS1 system, where RUNX1 is the main TF and ETS1 is the cooperator. Dotted vertical lines show the 0.2 false positive rate.

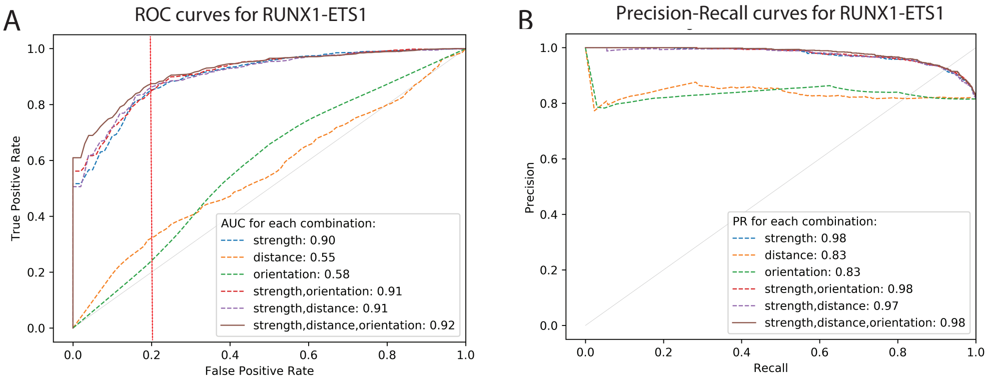

Fig. S14. Random Forest classification models can accurately distinguish between cooperative and independent binding for RUNX1-ETS1. (A) ROC curve. Dotted vertical line shows the 0.2 false positive rate. (B) Precision recall curve is also shown, to account for the small number of cooperative binding events in RUNX1-ETS1 data set. For each set of features, the false positive and true positive rates were computed by averaging over the 10 folds of a cross-validation test.

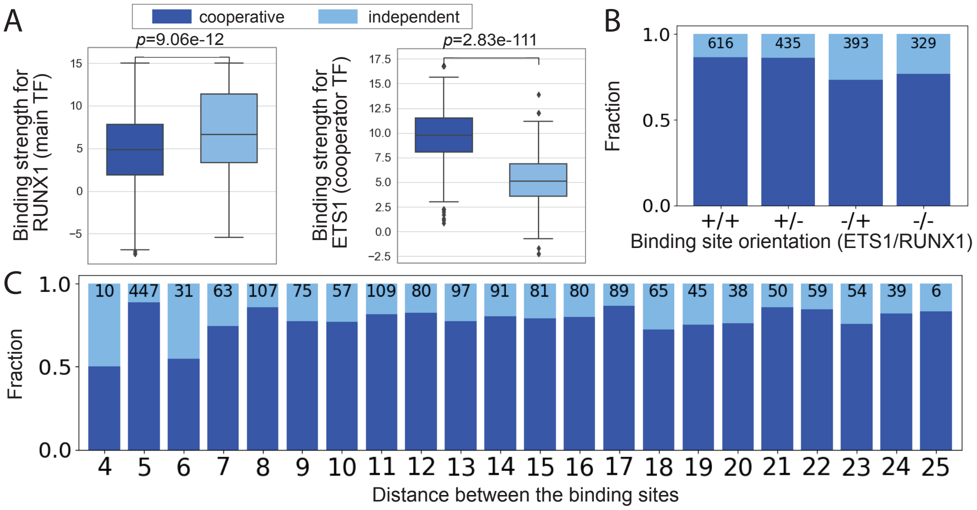

Fig. S15. Analyses of features used to train cooperative binding models on the RUNX1-ETS1 data. (A) Binding site score distributions for sequences with cooperative vs. independent binding. Y-axes show PWM scores. P-values were computed using the Mann-Whitney U test.(B,C) Fractions of probes bound cooperatively vs. independently, for different orientations and distances between the ETS1 and RUNX1 sites. Numbers above the barplots show the total number of sequences in each category.

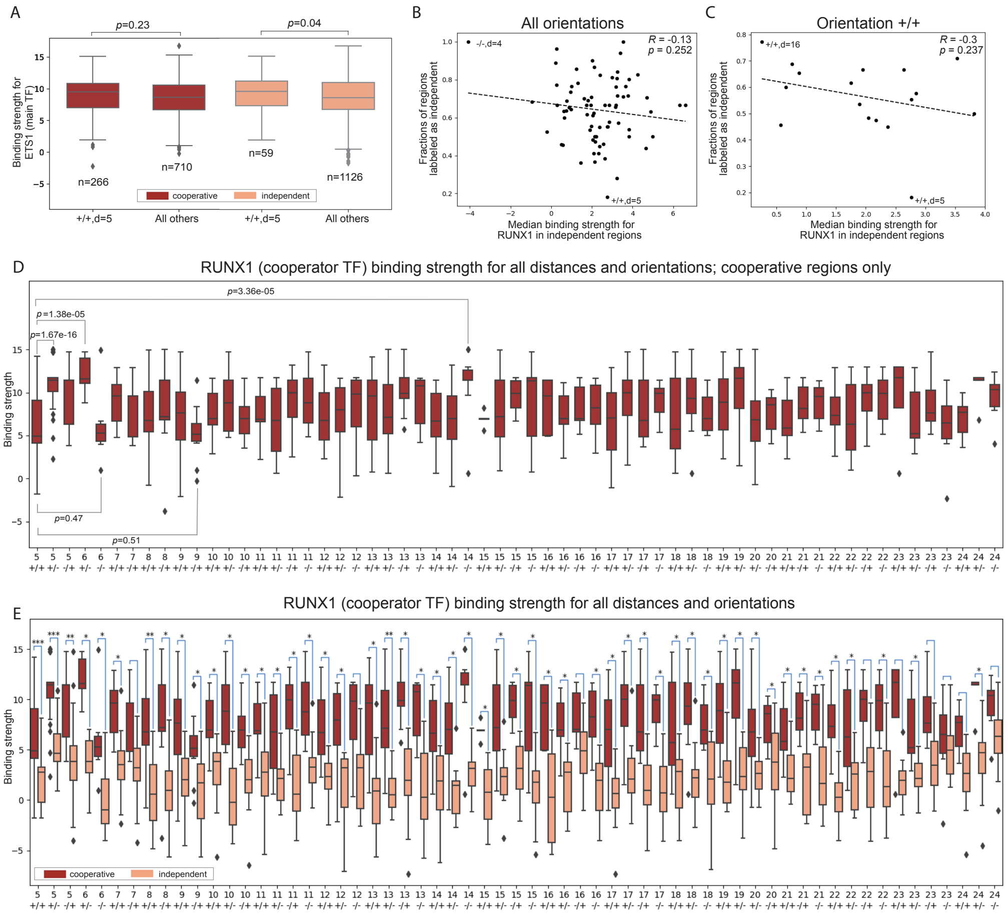

Fig. S16. (A) Binding strength of ETS1 for the sites in the ETS1-RUNX1 experiment for orientation +/+,distance=5 versus all other configurations. The numbers below each box plots show the number of genomic regions in each category. No significant difference was observed for the ETS1 binding strength. (B) RUNX1 binding strength in regions labeled as independent. The x-axis shows the median RUNX1 binding strength over all independent regions with a particular configuration, i.e. distance + orientation. The y-axis shows how "preferred" each configuration is for independent binding, assessed by the fraction of independent binding regions with that configuration. Only configurations with 5 or more regions in our DNA library are shown. (C) Same as (B) but showing only the +/+ orientation. (D) RUNX1 binding strength for all configurations in the cooperative regions. Only configurations with 5 or more regions in our DNA library are shown. Highlighted above the box plots are the top three configurations for which the RUNX1 strength is most different compared to (+/+,d=5). Highlighted below the box plots are the top two configurations for which the RUNX1 strength is most similar compared to (+/+,d=5); these two configurations, (-/-,d=6) and (-/+,d=9) also seem favourable for cooperativity, since cooperative binding occurs even when the cooperator site (here, RUNX1) is of low affinity. (E) Similar to panel (D), but for both cooperative and independent regions. We found that RUNX1 binding strength is always higher at the cooperative regions. The asterisks mark the significance of the difference in RUNX1 binding strength between cooperative and independence regions: *** : $p < 1e-10$; ** : $p < 1e-5$; * : $p < 1e-2$.

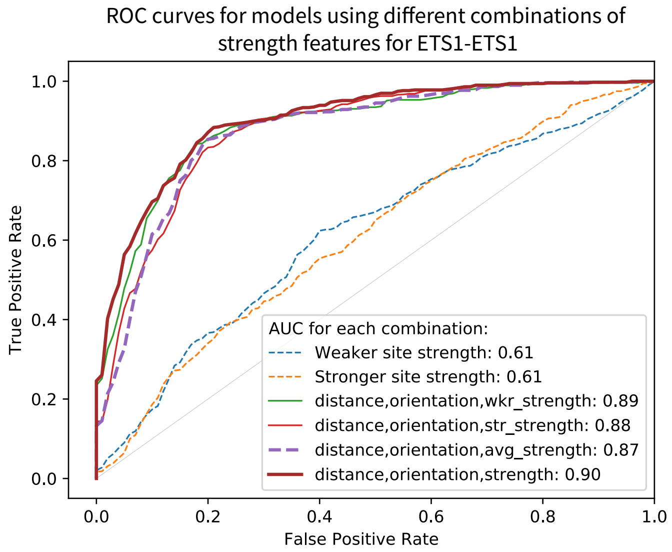

Fig. S17. Performance of Random Forest models trained separately on the binding site strength of either the weaker or the stronger of the two ETS1 sites, for the ETS1-ETS1 system.

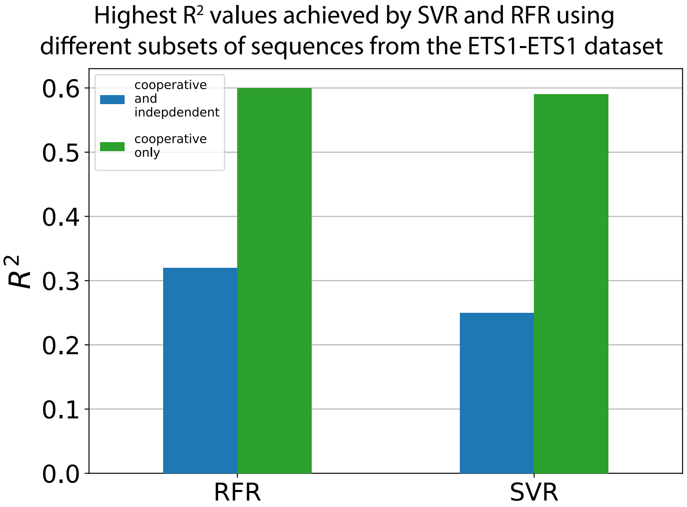

Fig. S18. The highest R^2 scores (from 5-fold cross-validation; computed over the hyperparameters specified in **Table S7A,B**) for RFR (left) and SVR (right) using both cooperative and independent sequences (blue), or only cooperative sequences (green).

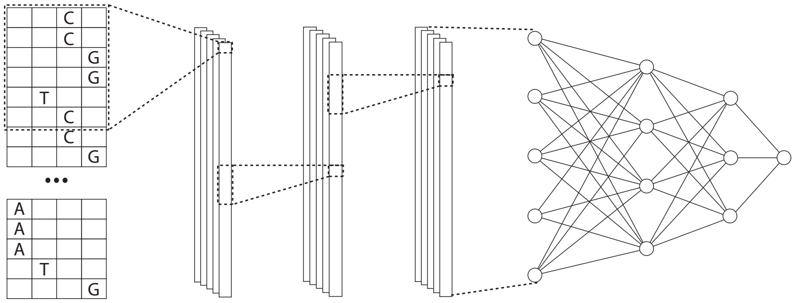

Fig. S19. Diagram showing the main features of the convolutional neural network. One-hot encoded sequences are fed into the network through a series of convolutional layers which extract patterned information from across the sequence. The feature maps learned in the convolutional layers are flattened and fed into a series of fully-connected layers, which output a numerical prediction for the cooperativity level.

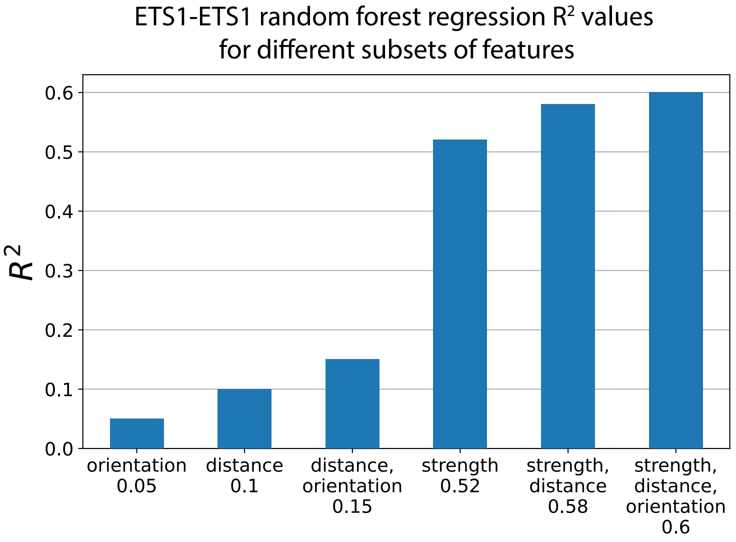

Fig. S20. R^2 values for random forest regression (RFR) models trained on the ETS1-ETS1 dataset using different subsets of features.

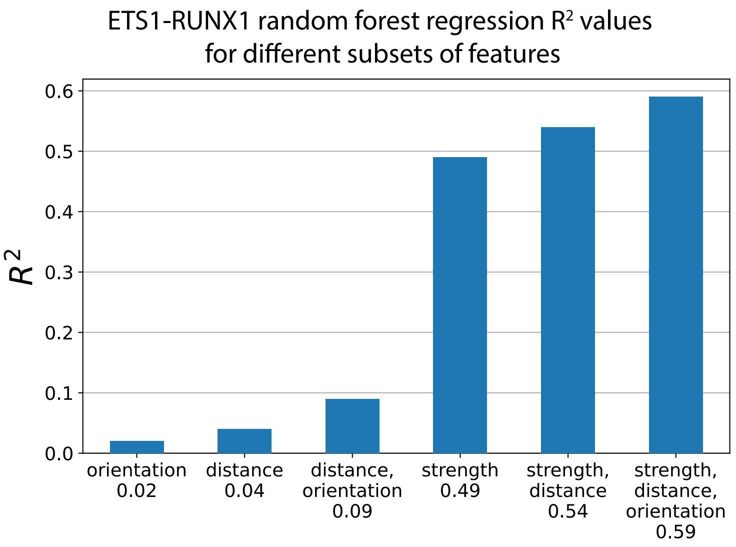

Fig. S21. R^2 values for random forest regression (RFR) models trained on the ETS1-RUNX1 dataset using different subsets of features.

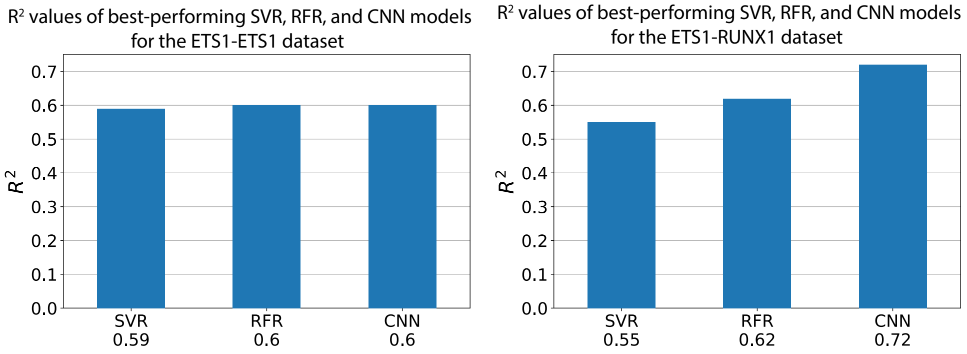

Fig. S22. Accuracy of the best regression models trained using SVR, RFR, or CNN algorithms for the ETS1-ETS1 system (left) and the ETS1-RUNX1 system (right). For each type of model, the plots show the highest R^2 achieved on the test sets in 5-fold cross-validation experiments.

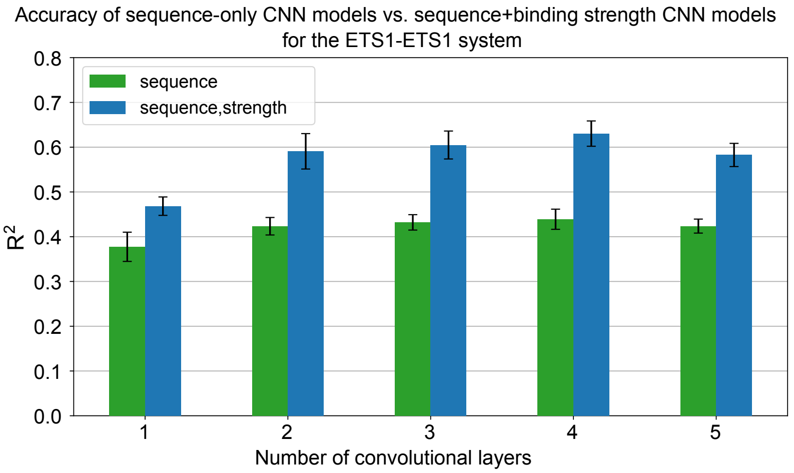

Fig. S23. Accuracy of sequence-only CNN models vs. sequence+binding strength CNN models for the ETS1-ETS1 system. For each number of layers, the best prediction accuracy (over the tested hyperparameters, **Table S7**) is shown for models that either include (blue) or do not include (green) input features reflecting the binding strengths of the two ETS1 binding sites. Error bars correspond to standard deviation of the five cross-validation R^2 values (Supplementary Methods).

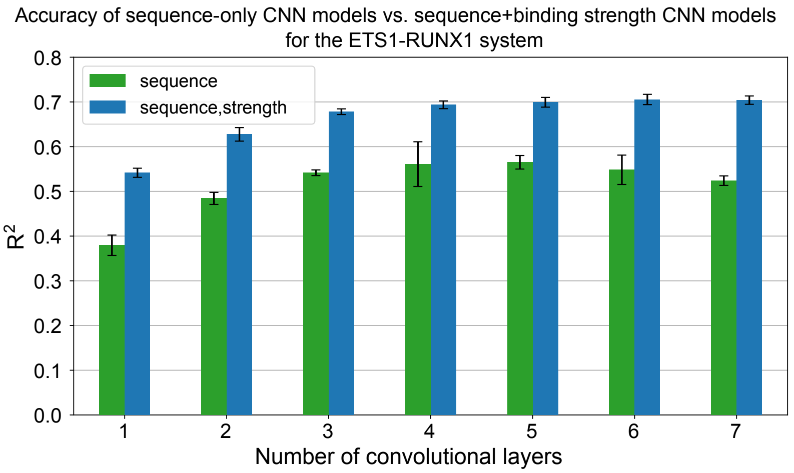

Fig. S24. Accuracy of sequence-only CNN models vs. sequence+binding strength CNN models for the ETS1-RUNX1 system. For each number of layers, the best prediction accuracy (over the tested hyperparameters, **Table S7**) is shown for models that either include (blue) or do not include (green) input features reflecting the binding strengths of the ETS1 and RUNX1 binding sites. Error bars correspond to standard deviation of the five cross-validation R^2 values (Supplementary Methods).

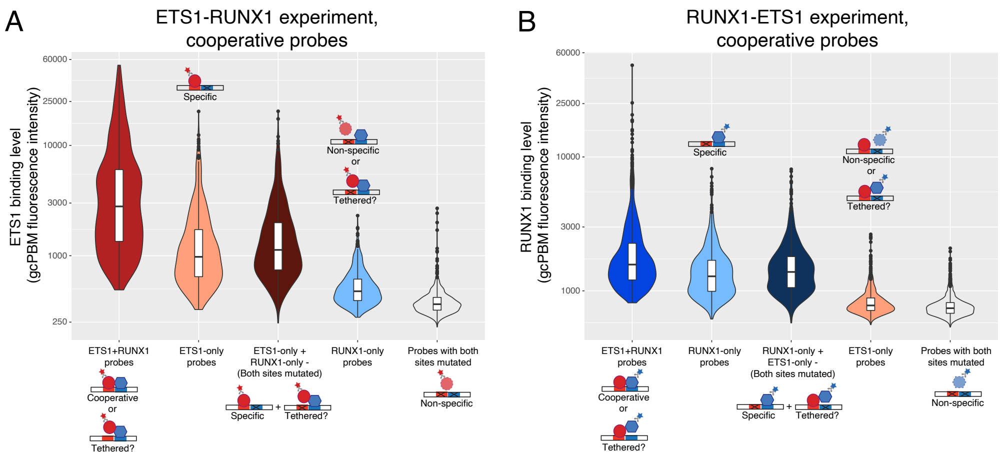

Fig. S25. TF binding levels at cooperative wild-type probes vs. mutated probes. (A) ETS1 binding levels at probes labeled as cooperative (Materials and Methods), as well mutated versions of these probes, as measured in the ETS1-RUNX1 experiment. The violin plots show the distributions of: ETS1 binding levels at wild-type cooperative probes, which contain binding sites for both ETS1 and RUNX1 (red); ETS1 binding levels at cooperative probes with the RUNX1 site mutated, i.e. ETS1-only probes (orange); ETS1 binding levels at cooperative probes with the ETS1 site mutated, i.e. RUNX1-only probes (light blue); ETS1 binding levels at cooperative probes with both sites mutated (empty violin); and the sum of ETS1 binding levels at ETS1-only and RUNX1-only probes, minus the binding level at probes with both sites mutated (dark red). All pairwise comparisons between the different sets are significant according to a Mann-Whitney U test ($p<2.2e-16$), which is not surprising given the large sample sizes. (B) Similar to panel A, but for RUNX1 binding levels as measured in the RUNX1-ETS1 experiment. The protein measured in each experiment is the one bound by the antibody: red = ETS1, blue = RUNX1. The designations of "Specific", "Non-specific", "Cooperative" and "Tethered" refer to DNA binding by the protein measured in each experiment.

5. SUPPLEMENTARY REFERENCES

REFERENCES

1. A. Paszke, S. Gross, S. Chintala, G. Chanan, E. Yang, Z. DeVito, Z. Lin, A. Desmaison, L. Antiga, and A. Lerer, "Automatic differentiation in pytorch," in *NIPS-W,* (2017).
2. D. P. Kingma and J. Ba, "Adam: A method for stochastic optimization," arXiv preprint arXiv:1412.6980 (2014).
3. F. Pedregosa, G. Varoquaux, A. Gramfort, V. Michel, B. Thirion, O. Grisel, M. Blondel, P. Prettenhofer, R. Weiss, V. Dubourg, J. Vanderplas, A. Passos, D. Cournapeau, M. Brucher, M. Perrot, and E. Duchesnay, "Scikit-learn: Machine learning in Python," J. Mach. Learn. Res. **12**, 2825–2830 (2011).
4. D. Johnson, A. Mortazavi, R. Myers, and B. Wold, "Genome-wide mapping of in vivo protein-DNA interactions," Science **316**, 1497–1502 (2007).
5. T. U. Consortium, "UniProt: the Universal Protein Knowledgebase in 2023," Nucleic Acids Res. **51**, D523–D531 (2022).
6. T. Shrivastava, K. Mino, N. D. Babayeva, O. I. Baranovskaya, A. Rizzino, and T. H. Tahirov, "Structural basis of Ets1 activation by Runx1," Leukemia **28**, 2040–2048 (2014).
7. J. Liu, C. A. Shively, and R. D. Mitra, "Quantitative analysis of transcription factor binding and expression using calling cards reporter arrays," Nucleic Acids Res **48**, e50 (2020).
8. Avsec, M. Weilert, A. Shrikumar, S. Krueger, A. Alexandari, K. Dalal, R. Fropf, C. McAnany, J. Gagneur, A. Kundaje, and J. Zeitlinger, "Base-resolution models of transcription-factor binding reveal soft motif syntax," Nat Genet. **53**, 354–366 (2021).
9. M. Tugrul, T. Paixao, N. H. Barton, and G. Tkacik, "Dynamics of Transcription Factor Binding Site Evolution," PLoS Genet. **11**, e1005639 (2015).
10. D. Ezer, N. R. Zabet, and B. Adryan, "Homotypic clusters of transcription factor binding sites: A model system for understanding the physical mechanics of gene expression," Comput. Struct Biotechnol J **10**, 63–69 (2014).
11. D. Wotton, J. Ghysdael, S. Wang, N. A. Speck, and M. J. Owen, "Cooperative binding of Ets-1 and core binding factor to DNA," Mol Cell Biol **14**, 840–850 (1994).
12. W. Sun, B. J. Graves, and N. A. Speck, "Transactivation of the Moloney murine leukemia virus and T-cell receptor beta-chain enhancers by cbf and ets requires intact binding sites for both proteins," J Virol **69**, 4941–4949 (1995).
13. T. Zhou, L. Yang, Y. Lu, I. Dror, A. C. Dantas Machado, T. Ghane, R. Di Felice, and R. Rohs, "DNAshape: a method for the high-throughput prediction of DNA structural features on a genomic scale," Nucleic Acids Res. **41**, 56–62 (2013).
14. J. E. Moore, M. J. Purcaro, H. E. Pratt, C. B. Epstein, N. Shoresh, J. Adrian, T. Kawli, C. A. Davis, A. Dobin, R. Kaul, J. Halow, E. L. Van Nostrand, P. Freese, D. U. Gorkin, Y. Shen, Y. He, M. Mackiewicz, F. Pauli-Behn, B. A. Williams, A. Mortazavi, C. A. Keller, X. O. Zhang, S. I. Elhajjajy, J. Huey, D. E. Dickel, V. Snetkova, X. Wei, X. Wang, J. C. Rivera-Mulia, J. Rozowsky, J. Zhang, S. B. Chhetri, J. Zhang, A. Victorsen, K. P. White, A. Visel, G. W. Yeo, C. B. Burge, E. Lécuyer, D. M. Gilbert, J. Dekker, J. Rinn, E. M. Mendenhall, J. R. Ecker, M. Kellis, R. J. Klein, W. S. Noble, A. Kundaje, R. Guigó, P. J. Farnham, J. M. Cherry, R. M. Myers, B. Ren, B. R. Graveley, M. B. Gerstein, L. A. Pennacchio, M. P. Snyder, B. E. Bernstein, B. Wold, R. C. Hardison, T. R. Gingeras, J. A. Stamatoyannopoulos, and Z. Weng, "Expanded encyclopaedias of DNA elements in the human and mouse genomes," Nature **583**, 699–710 (2020).
15. T. Liu, J. A. Ortiz, L. Taing, C. A. Meyer, B. Lee, Y. Zhang, H. Shin, S. S. Wong, J. Ma, Y. Lei, U. J. Pape, M. Poidinger, Y. Chen, K. Yeung, M. Brown, Y. Turpaz, and X. S. Liu, "Cistrome: an integrative platform for transcriptional regulation studies," Genome Biol. **12**, R83 (2011).

16. M. F. Berger, A. A. Philippakis, A. M. Qureshi, F. S. He, P. W. Estep, and M. L. Bulyk, "Compact, universal DNA microarrays to comprehensively determine transcription-factor binding site specificities," Nat. Biotechnol. **24**, 1429–1435 (2006).
17. M. F. Berger and M. L. Bulyk, "Universal protein-binding microarrays for the comprehensive characterization of the DNA-binding specificities of transcription factors," Nat Protoc **4**, 393–411 (2009).
